# Environmentally friendly domino multicomponent strategy for the synthesis of pyrroloquinolinone hybrid heterocycles†

**DOI:** 10.1039/d2ra02851d

**Published:** 2022-05-25

**Authors:** Suresh Mani, Rajesh Raju, Raghavacharry Raghunathan, Natarajan Arumugam, Abdulrahman I. Almansour, Raju Suresh Kumar, Karthikeyan Perumal

**Affiliations:** Department of Organic Chemistry, University of Madras, Guindy Campus Chennai 600025 India vrajurajesh@gmail.com; Department of Chemistry, College of Science, King Saud University P. O. Box 2455 Riyadh 11451 Saudi Arabia; Department of Chemistry and Biochemistry, The Ohio State University 151 W. Woodruff Ave Columbus OH 43210 USA

## Abstract

An efficient and elegant assembly of pyrene/aryl fused pyrrolo[2,3-*b*]quinolinone and pyrrolizino[3,2-*b*]quinolinone hybrid heterocycles was achieved *via* a domino multicomponent reaction strategy using a solid state melt reaction (SSMR) condition. The 1,3-dipole component was generated *in situ* from *N*-methylgylcine/l-proline and isatin, while the Baylis–Hillman adduct prepared from pyrene-1-carbaldehyde and various benzaldehydes is used as the dipolarophile. The domino protocol comprises 1,3-dipolar cycloaddition and a consequent double annulation reaction process. The advantages of this cascade protocol include environmentally friendly conditions, the avoidance of toxic organic solvents, simple work-up and good to excellent product yields.

## Abstract 2

An efficient and elegant assembly of pyrene/aryl fused pyrrolo[2,3-*b*]quinolinone and pyrrolizino[3,2-*b*]quinolinone hybrid heterocycles was achieved *via* a domino multicomponent reaction strategy using a solid state melt reaction (SSMR) condition.

## Graphical abstract

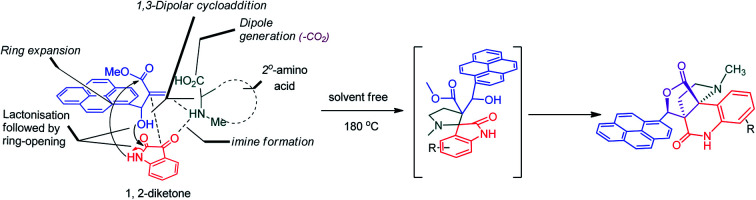

## Introduction

Domino multicomponent reaction is an attractive synthetic methodology that creates several transformations with notable advantages such as high reaction efficiency, convergence, elegance, cost savings, simple automation and minimization of synthetic work up steps.^1^ This protocol obviates the isolation and purification steps which leads to an improvement in the overall yield compared to classical multistep synthetic transformations in a single pot manipulation and represents an attractive synthetic method for building hybrid heterocyclic architectures.^2^ Besides, this protocol is well suited for the production of structurally diverse hybrid heterocycles with multiple stereogenic centers and biologically relevant molecules.^3^

Domino multicomponent reactions involving 1,3-dipolar cycloadditions are attractive in this connection as this reaction allows for the efficient assembly of biologically relevant five-membered heterocycles. This synthetic protocol has recently received considerable attention in organic synthesis.^4–7^ In particular, the synthesis of the pyrrolidine unit by the reaction of alkenes with azomethine ylides gains importance because of their occurrence in numerous bioactive natural products.^8–10^ In this context, we recently reported the synthesis of structurally unusual hybrid heterocycles comprising spiropyrrolidine units^11^ and pyrroloquinolinone analogs prepared from Baylis–Hillman adducts *via* the multicomponent 1,3-dipolar cycloaddition and domino reaction sequence.^12^ Spiropyrrolidine analogues possess an interesting biological profiles including antimicrobial,^13^ anti-cancer^14^ and anti-Alzheimer^15,16^ activities. Pyrroloquinolinone analogs are found to be an essential component of many natural products such as melodinus alkaloids, (+) scandin, (+) meloscine, meloscandinone^17,18^ which are used in traditional Chinese folkmedicine to treat meningitis in children and rheumatic heart disease.

**Fig. 1:**
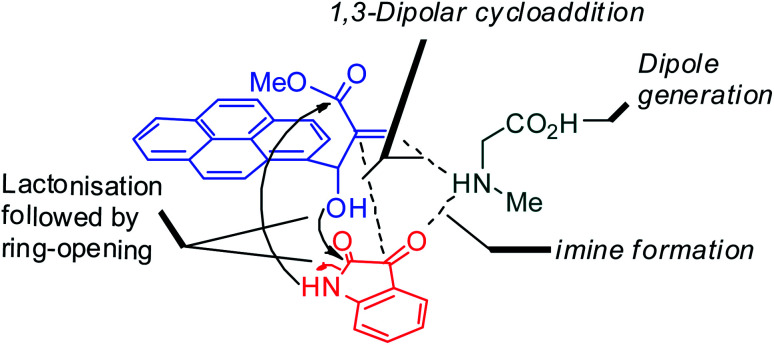
Synthetic strategy of pyrroloquinolinones.

With an aim to construct novel heterocycles comprising pyrrolo [3,2-*c*]quinolino/pyrrolizino [2,3-*c*] quinolinone, we have now investigated the synthetic utility of Baylis–Hillman adducts as dipolarophiles through sustainable green domino protocol that involves a decarboxylative 1,3-dipolar cycloaddition^19–21^ followed by a double annulation *via* sequential lactonization and lactamization reactions. Our synthetic strategy employed for the synthesis of pyrroloquinoline heterocycles is shown in Fig. 1.

## Results and discussion

**Scheme 1:**
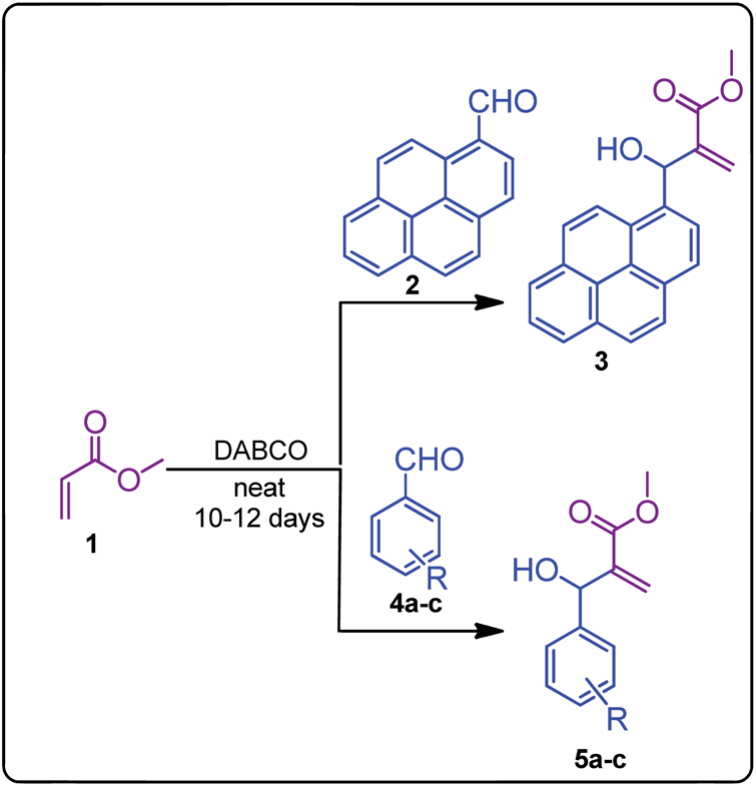
Synthesis of Baylis–Hillman adducts.

The structurally unexplored new class of highly functionalized starting precursors *viz.* 3-(1,8-dihydropyren-1-yl)-2-methylenebutanoate 3 and various substituted methyl 2-(hydroxy(phenyl)methyl)acrylate 5 were required for the synthesis of pyrroloquinolinones, which were synthesized from pyrene-1-carbaldehyde 2 and various substituted benzaldehydes 4 by the Baylis–Hillman reaction (Scheme 1). Incidentally, pyrene is important polycyclic aromatic ring system that has been shown diverse biological activity including cytotoxicity against human cancer cell lines.^22^

**Scheme 2:**
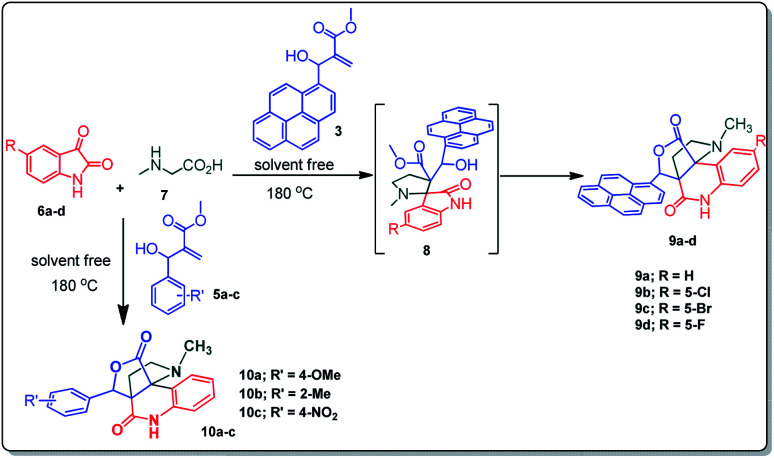
Synthesis of pyrroloquinolinone hybrids 9a–d and 10a–c.

The domino multicomponent reaction was carried out by the reaction of isatin 6, sarcosine 7 and the Balylis–Hillman adduct 3 under solvent free melting conditions to furnish the unexpected pyrene tethered pyrroloquinolinone hybrids 9a–d in excellent yields (Scheme 2). Firstly, the domino reaction was examined with various solvent systems such as CH_3_CN, toluene, xylene and dioxane as summarized in Table 1. However, even after a longer reaction time under these solvent conditions, we did not obtain any fruitful results, but instead ended up with an inseparable mixture of the products. Ultimately, the domino reaction was carried out under solid state melt reaction conditions. Thus, in a typical reaction, the starting substrate 3, 6a and 7 in a round bottom flask at 180 °C under solvent free conditions afforded the desired product 9a in 93% yield without column purification. It was found that 180 °C was the optimum temperature for this solid-state melt reaction in terms of both yield and time. The reaction was also performed with Baylis–Hillman adduct 5a–c under the above optimized reaction conditions, which afforded 10 in good to excellent yields. It is pertinent to note that the compounds synthesized are racemates which has been indicated in Table 2.

**Table 1:** Optimization of reaction conditions for the synthesis of compound 9a

Entry	Solvents	*T* (°C)	Time	Yield^a^ (%)
1	Acetonitrile	85	12 h	—
2	Toluene	110	12 h	—
3	Xylene	120	12 h	—
4	Dioxane	100	12 h	—
5	None^b^	100	1 h	—
6	None^b^	130	1 h	15
7	None^b^	160	1 h	63
8	None^b^	180	10 min	93

a Isolated yield of the products.

b Reactants melted at the mentioned temperature.

**Table 2:** Synthesis of aryl fused polycyclic pyrroloquinolinone derivatives

Entry	Dipolarophile	1,2-Diketone	Sec-amino acid	Mixture of stereoisomers^a^	Yield^b^
1	3	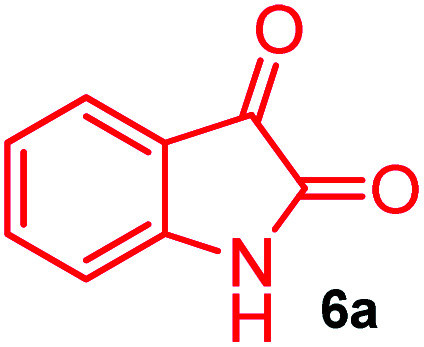	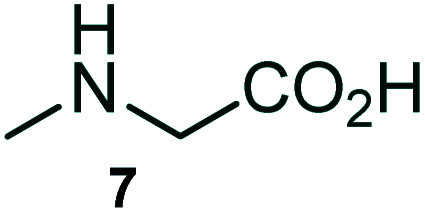	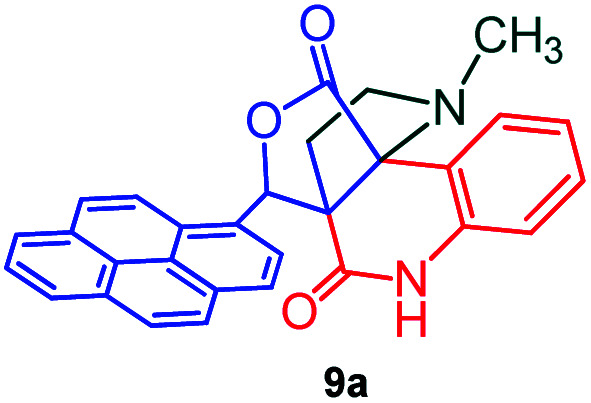	93
2	3	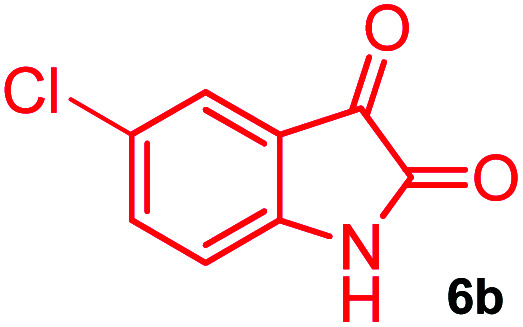	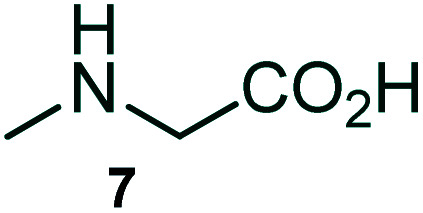	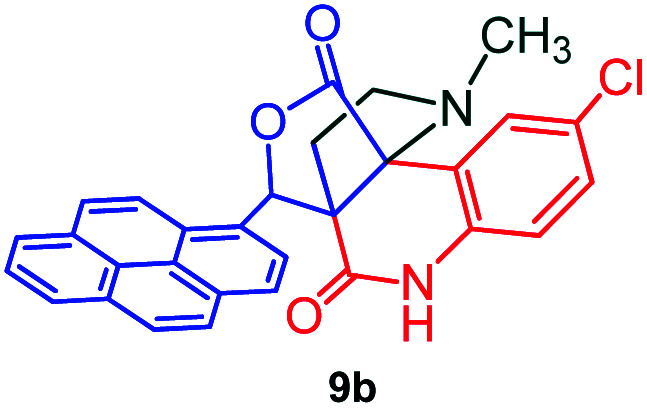	96
3	3	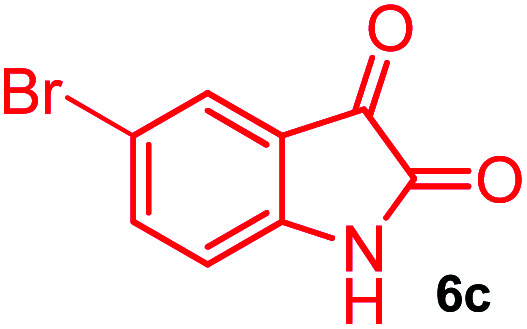	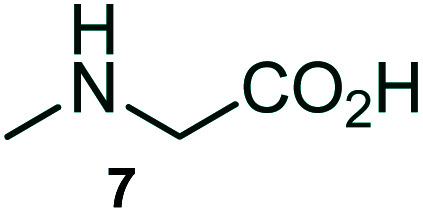	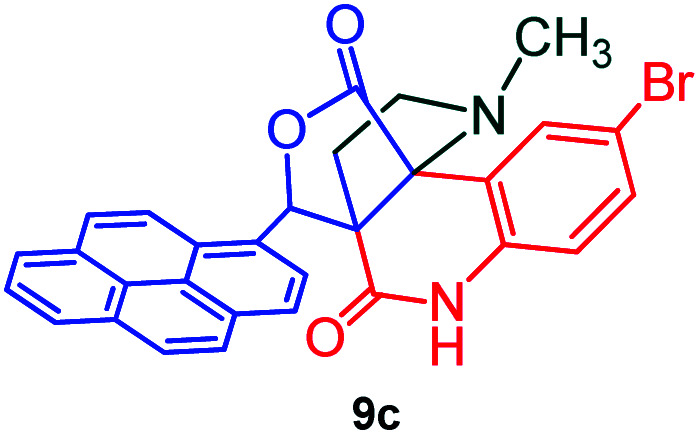	95
4	3	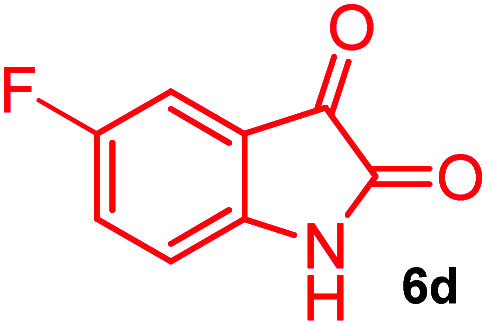	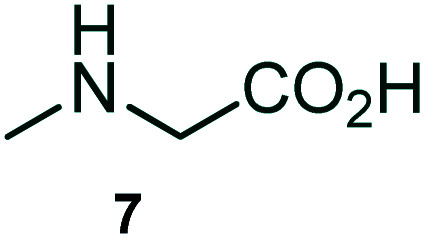	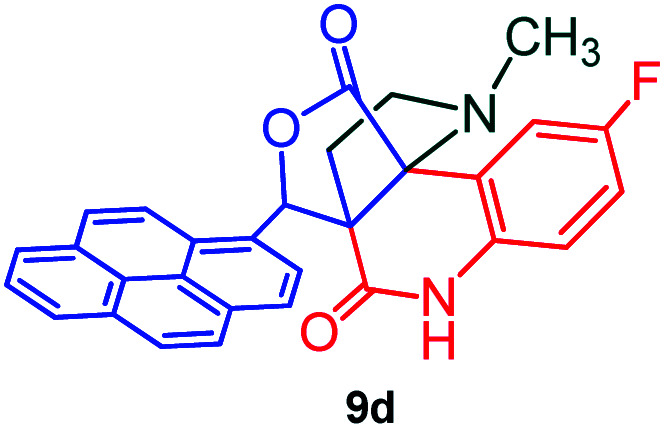	91
5	5a	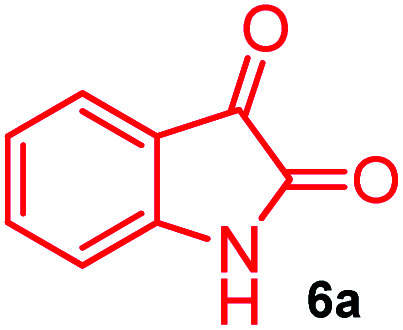	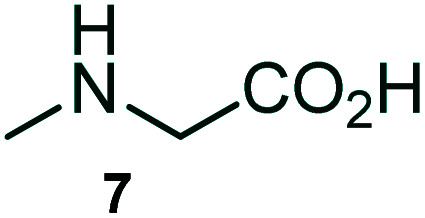	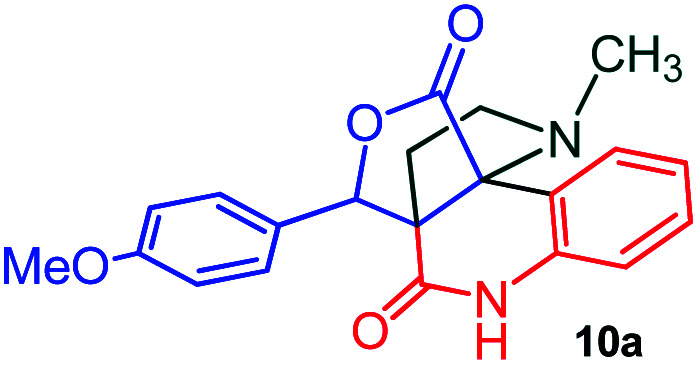	92
6	5b	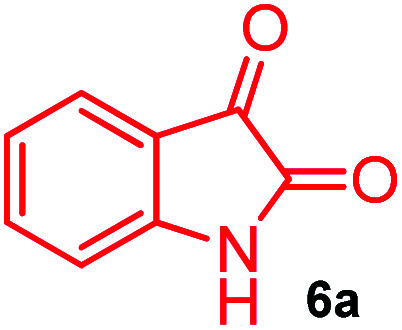	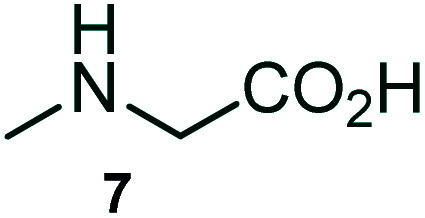	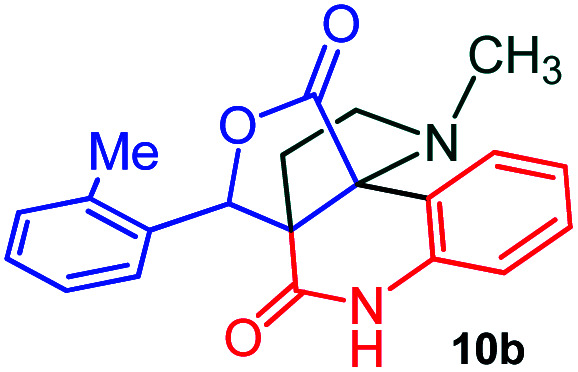	93
7	5c	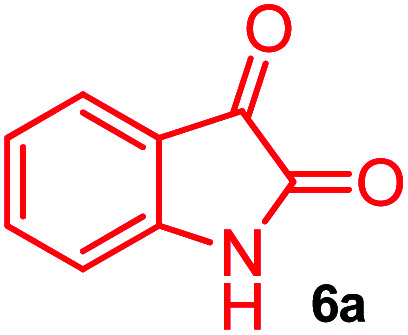	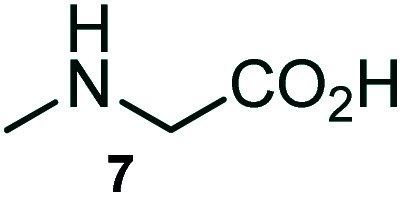	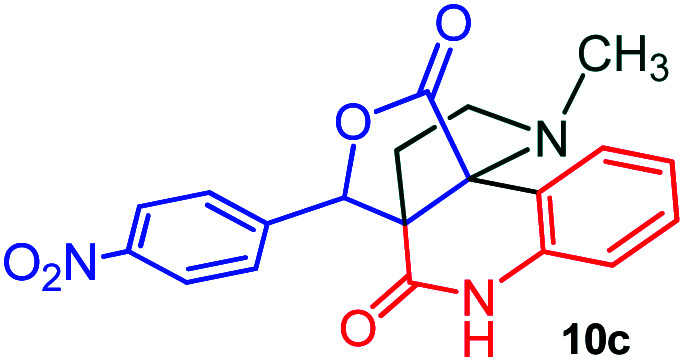	87
8	3	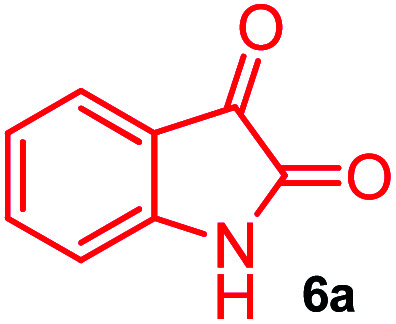	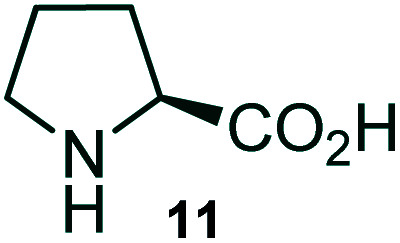	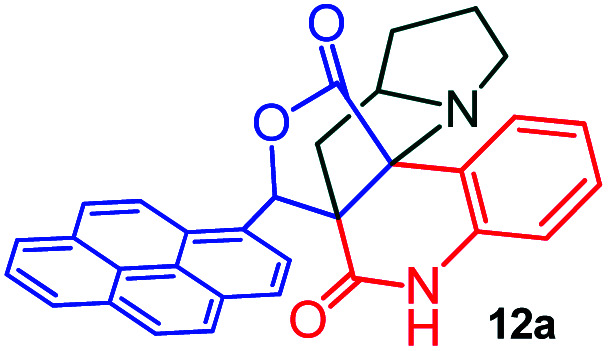	94
9	3	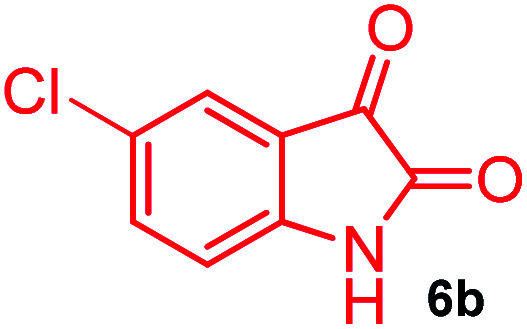	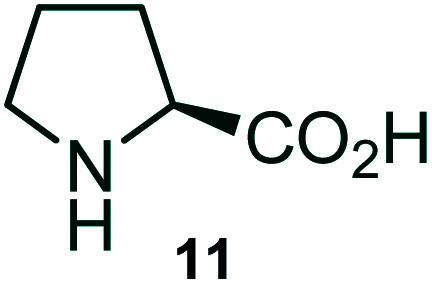	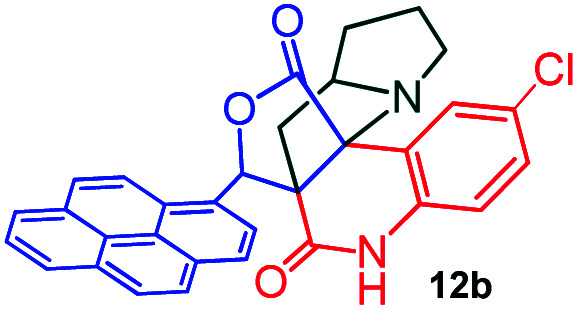	91
10	3	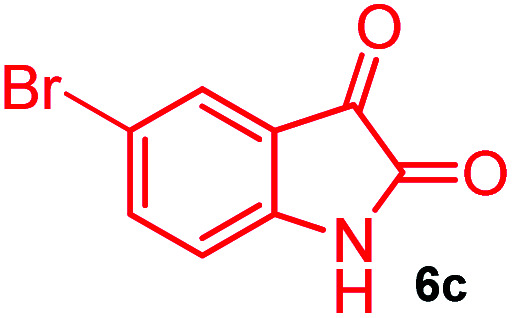	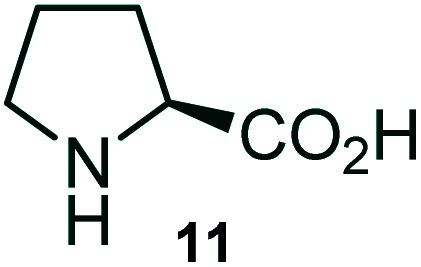	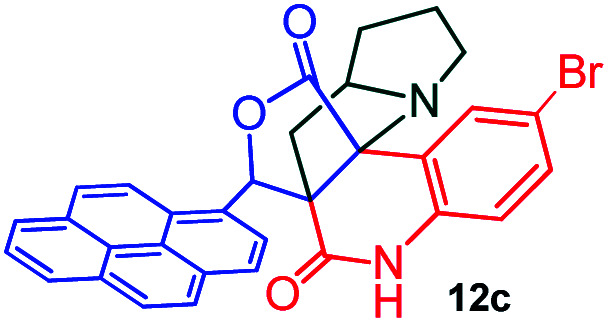	92

a Isolated products are mixture of stereoisomers.

b Yield of isolated product after column chromatography.

**Fig. 2:**
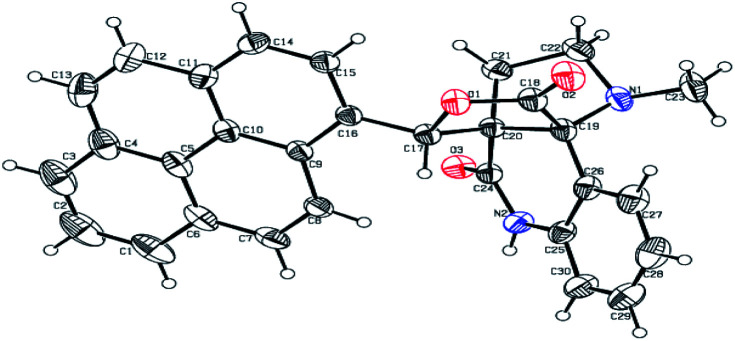
ORTEP diagram of compound 9a.

The formation of the pyrroloquinoline 9a was unambiguously determined by spectroscopic data. In the ^1^H and ^13^C NMR spectrum of 9a, interestingly, the absence of the methoxy(–OCH_3_) and the –OH groups confirm the formation of the unusual cyclized product. In the ^1^H NMR spectrum, the hydrogens of the –NCH_3_ unit and –NH of lactam resonated as singlet at *δ* 2.63 and 10.78 ppm respectively. The –NCH_2_ hydrogens of the pyrrolidine ring exhibited as multiplets at *δ* 2.84 and *δ* 3.07. In the ^13^C NMR spectra, the carbon signals at *δ* 178.9 and 173.2 ppm were ascribable to quinolinone and lactone ring carbonyl carbons, respectively. The carbon signal at *δ* 39.3 ppm was assignable to –NCH_3_ carbon and the two carbon signals at *δ* 64.3 and 78.5 ppm were due to two bridged quaternary carbons, respectively. The two methylene groups appeared in negative region at *δ* 30.9 and 57.3 ppm which was further confirmed by DEPT-135 NMR spectrum. The observed chemical shifts are in agreement with the structure of 9a. Finally, compound 9a was unambiguously determined by single crystal X-ray crystallographic analysis as shown in Fig. 2.^23^

**Scheme 3:**
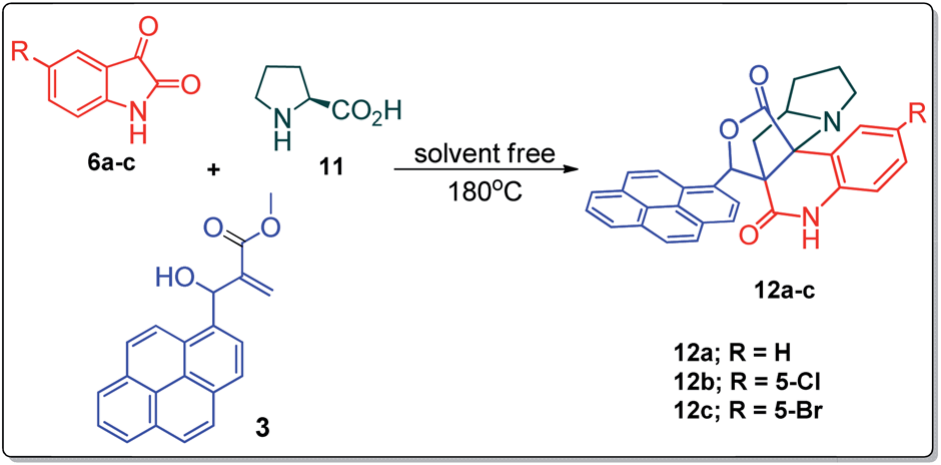
Synthesis of pyrene fused pyrrolizidinoquinolines

Further, we have extended the scope of this multicomponent cascade transformation, by using cyclic amino acid namely l-proline, as described in Scheme 3. Thus, the reaction of non-stabilized azomethine ylides generated *in situ* from isatin 6a–c and l-proline 11 with the Baylis–Hillman adduct 3 under the optimized conditions afforded pyrrolizinoquinolinone hybrids 12a–c in good yields. The structure of pyrrolizinoquinoline was elucidated by spectroscopic data.

**Scheme 4:**
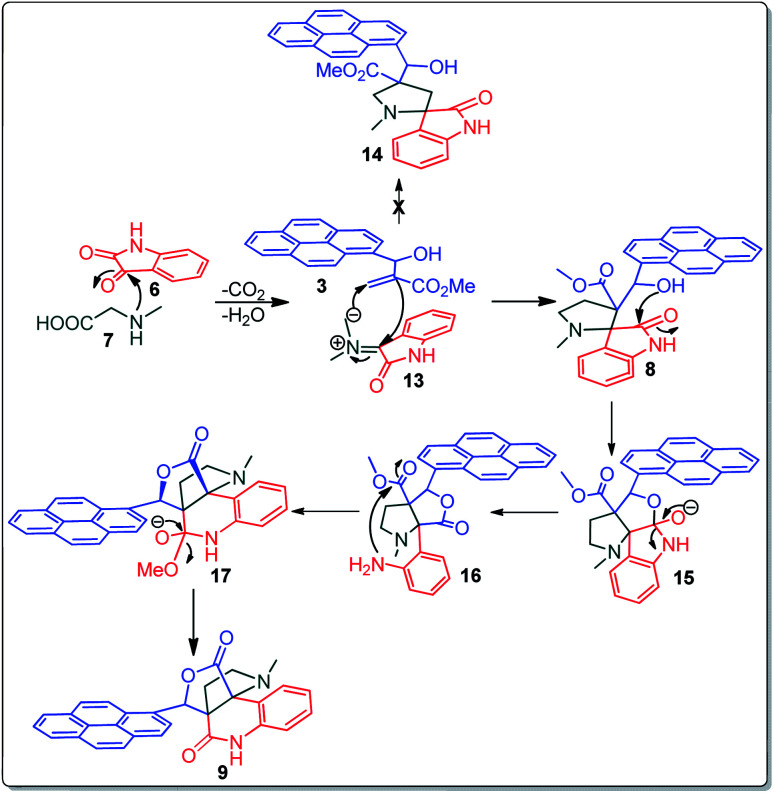
Plausible reaction pathway for the formation of 9.

A likely mechanistic pathway for the assembly of pyrroloquinolinone hybrids *via* the multicomponent domino reaction is shown in Scheme 4. Initially, the reaction of isatin 6 and sarcosine 7 forms the non-stabilized 1,3-dipole component 13 through spontaneous dehydration and decarboxylation. Subsequently, the reaction of dipole component 13 with the dipolarophile 3 can take place through spiro intermediate 14 or 8 to furnish 9. Thus, the exclusive formation of spiro intermediate 8 in the cycloaddition process shows that compound 8 is favoured over compound 14. Subsequently, the hydroxyl group of spiro intermediate 8 underwent an intramolecular nucleophilic attack on the carbonyl of the adjacent oxindole ring, leading to the formation of the intermediate 15, which in turn led to an aniline intermediate 16 through stabilization of the oxygen anion. Finally, the amino group was cyclized to the final product 9 by nucleophilic attack on ester carbonyl followed by elimination of the methoxy group. Formation of an unusual product by two C–N, three C–C bonds that have three stereogenic center in single-pot synthetic operation.

## Conclusions

In summary, we have successfully synthesized structurally interesting unexplored new class of pyrene/aryl derived pyrroloquinolinone and pyrene derived pyrrolizinoquinoline hybrid heterocycles employing domino multicomponent reaction strategy under eco-friendly solid-state melt reaction conditions. Besides, the starting precursor, the electron deficient pyrene alkene, was synthesized for the first time in excellent yield through Baylis–Hillman reaction. The domino reaction involves 1,3-dipolar cycloaddition followed by double annulation cascade reaction process, the product generates up to three stereocenter *via* two C–N and three C–C bonds. It is noteworthy to mention that this domino reaction was performed under solvent and catalyst free conditions, which is very attractive from green chemistry perspective.

## Experimental

### General considerations

^1^H and ^13^C NMR spectra were determined on a BRUKER 300 MHz NMR instrument in chloroform-d_6_ solvent with trimethyl silane (TMS) as a reference standard. High resolution mass spectra were obtained by ESI using Orbitrap elite mass spectrometer. C, H and N analyses were performed using PerkinElmer CHNS 2400B instrument. The X-ray diffraction analysis was determined on a Bruker (2008) SMART APEX II detector diffractometer. Silica gel (mesh size 100–200) was used for column chromatography and hexane and ethyl acetate as eluent. The reaction was monitoring by thin layer chromatography (TLC) developed on 25 mm silica gel-G (ACME) coated glass plates and monitoring by iodine.

### General procedure for synthesis of BAH, 3/5

A mixture of methyl acrylate 1 (1.5 mmol), pyrene-1-carbaldehyde 2 (1.0 mmol)/substituted benzaldehydes 4 (1.0 mmol) and DABCO (0.1 mmol) were allowed to stand at ambient temperature for 12–15 days. Upon completion of the reaction as evidenced by TLC analysis, the reaction mixture was diluted with ethyl acetate (20 mL) and washed successively with 2 N HCl solution, H_2_O and aqueous sodium bicarbonate solution. Organic solvent was dried over anhydrous Na_2_SO_4_ and evaporated under vaccuo. The residue was purified using column chromatography using hexane : EtOAc (8 : 2 v/v) to afford the alcohol 3/5.

#### Methyl 2-(hydroxy(pyren-1-yl)methyl)acrylate 3

^1^H NMR (300 MHz, CDCl3): *δ* 3.72 (s, –OCH_3_, 3H), 5.56 (s, 1H), 6.35 (s, 1H), 6.62 (s, 1H), 7.94–8.23 (m, 9H). ^13^C NMR (75 MHz, CDCl_3_): 52.11, 69.63, 123.0, 124.5, 124.7, 124.9, 125.2, 125.3, 125.9, 127.1, 127.4, 127.5, 127.8, 128.2, 130.6, 131.1, 131.3, 133.9, 142.2, 167.2 ppm. Mass: *m*/*z* 316.15 (M^+^).

### General procedure for synthesis of fused pyrrolo[3,2-*c*] quinolinone hybrids, 9/10

A mixture of BHA 3/5 (1 mmol), isatin 6 (1.1 mmol) and sarcosine 7 (1.1 mmol) was placed in a flask and melted at 180 °C, the completion of the reaction as confirmed by TLC. After the disappearance of the starting materials, the crude product was recrystallized with mixture of 5 mL ethyl acetate and hexane (ratio 1 : 4), successfully gave the pure desired product 9/10 as a solid.

#### (3aR*,9bR*,12R*)-1-Methyl-12-(pyren-1-yl)-2,3-dihydro-1*H*-3*a*,9*b*-(methanooxymethano)pyrrolo[3,2-*c*]quinoline-4,10(5*H*)-dione, 9a

Colorless solid (93%), Mp: 246–248 °C, IR (KBr): 1709, 1742 cm^−1^; ^1^H NMR (300 MHz, CDCl_3_ + DMSO-d_6_): *δ* 2.37–2.47 (m, 2H), 2.63 (s, –NCH_3_, 3H), 2.78–2.90 (m, 1H), 3.07–3.09 (m, 1H), 6.53 (s, 1H), 7.16 (t, *J* = 7.5, 6.6 Hz, 1H), 7.36–7.41 (m, 1H), 7.80 (d, *J* = 7.8 Hz, 1H), 7.95–8.22 (m, 9H), 10.78 (s, 1H, N–H). ^13^C NMR (75 MHz, CDCl_3_ + DMSO-d_6_): 31.0, 39.3, 57.3, 64.3, 78.5, 118.9, 121.6, 127.1, 127.9, 129.2, 129.3, 129.4, 130.0, 130.4, 130.9, 131.6, 132.0, 132.4, 132.8, 133.3, 134.4, 135.1, 135.5, 135.9, 136.3, 142.7, 173.2, 178.9 ppm. Mass: *m*/*z* 458.50 (M^+^). Anal. calcd. For C_30_H_22_N_2_O_3_: C, 78.59, H, 4.84, N, 6.11%; found: C, 78.61, H, 4.81, N, 6.14%.

#### (3aR*,9bR*,12R*)-8-Chloro-1-methyl-12-(pyren-1-yl)-2,3-dihydro-1*H*-3*a*,9*b*-(methanooxymethano)pyrrolo[3,2-*c*]quinoline-4,10(5*H*)-dione, 9b

Colorless solid (96%), Mp: 252–254 °C, IR (KBr): 1710, 1739 cm^−1^; ^1^H NMR (300 MHz, CDCl_3_ + DMSO-d_6_): *δ* 2.14–2.22 (m, 1H), 2.34–2.40 (m, 1H), 2.56 (s, −NCH_3_, 3H), 2.72 (dd, *J* = 8.7, 9.0 Hz, 1H), 3.02 (dd, *J* = 6.0, 6.3 Hz, 1H), 6.73 (s, 1H), 7.30 (d, *J* = 8.4 Hz, 1H), 7.52 (d, *J* = 8.7 Hz, 1H), 7.66 (s, 1H), 8.03–8.37 (m, 9H), 11.32 (s, 1H, N–H). ^13^C NMR (75 MHz, CDCl_3_ + DMSO-d_6_): 26.0, 33.9, 52.0, 58.6, 72.6, 76.0, 115.7, 118.2, 122.5, 123.6, 123.8, 124.2, 124.5, 125.2, 125.5, 126.2, 126.5, 126.9, 127.0, 127.2, 127.9, 128.1, 128.4, 129.9, 130.4, 130.6, 131.0, 137.1, 167.5, 173.2 ppm. Mass: *m*/*z* 492.16 (M^+^). Anal. calcd. For C_30_H_21_ClN_2_O_3_: C, 73.09, H, 4.29, N, 5.68%; found: C, 73.11, H, 4.27, N, 5.70%.

#### (3aR*,9bR*,12R*)-1-Methyl-12-(pyren-1-yl)-2,3-dihydro-1*H*-3*a*,9*b*-(methanooxymethano)pyrrolo[3,2-*c*]quinoline-4,10(5*H*)-dione, 9c

Colorless solid (95%), Mp: 261–263 °C, IR (KBr): 1707, 1735 cm^−1^; ^1^H NMR (300 MHz, DMSO-d_6_): *δ* 2.04–2.12 (m, 1H), 2.28–2.37 (m, 1H), 2.50 (s, –NCH_3_, 3H), 2.68–2.77 (m, 1H), 2.99–3.04 (m, 1H), 6.87 (s, 1H), 7.23 (d, *J* = 8.4 Hz, 1H), 7.73 (d, *J* = 8.1 Hz, 2H), 8.04–8.42 (m, 9H), 11.34 (s, 1H, N–H). ^13^C NMR (75 MHz, DMSO-d_6_): 26.1, 33.9, 52.0, 58.6, 72.6, 76.0, 113.9, 116.2, 118.7, 122.9, 123.5, 123.7, 124.4, 124.8, 125.4, 125.7, 126.4, 127.1, 128.0, 128.2, 129.9, 130.7, 130.9, 131.1, 133.4, 137.6, 167.6, 173.5 ppm. Mass: *m*/*z* 536.12 (M^+^).Anal. calcd. For C_30_H_21_BrN_2_O_3_: C, 67.05, H, 3.94, N, 5.21%; found: C, 73.11, H, 4.27, N, 5.70%.

#### (3aR*,9bR*,12R*)8-Fluoro-1-methyl-12-(pyren-1-yl)-2,3-dihydro-1*H*-3*a*,9*b*-(methanooxymethano)pyrrolo[3,2-*c*]quinoline-4,10(5*H*)-dione, 9d

Colorless solid (91%), Mp: 242–244 °C, IR (KBr): 1712, 1741 cm^−1^; ^1^H NMR (300 MHz, CDCl_3_ + DMSO-d_6_): *δ* 2.16–2.24 (m, 1H), 2.33–2.40 (m, 1H), 2.52 (s, –NCH_3_, 3H), 2.73 (dd, *J* = 8.4, 9.0 Hz, 1H), 3.01–3.06 (m, 1H), 6.67 (s, 1H), 7.31 (d, *J* = 6.3 Hz, 2H), 7.43 (d, *J* = 8.7 Hz, 1H), 8.04–8.35 (m, 9H), 11.22 (s, 1H, N–H). ^13^C NMR (75 MHz, CDCl_3_ + DMSO-d_6_): 23.6, 31.6, 49.7, 56.1, 70.4, 73.7, 76.2, 112.8, 112.9, 113.0, 113.1, 114.9, 115.2, 115.6, 115.7, 120.1, 121.2, 121.5, 121.8, 122.1, 122.8, 123.1, 123.7, 124.5, 124.6, 124.8, 125.4, 125.8, 127.5, 128.3, 128.6, 132.2, 165.0, 170.9 ppm. Mass: *m*/*z* 476.18 (M^+^). Anal. calcd. For C_30_H_21_FN_2_O_3_: C, 75.62, H, 4.44, N, 5.88% found: C, 75.65, H, 4.46, N, 5.85%.

#### (3aR*,9bR*,12R*)-1-Methyl-12-(4-methoxyphenyl)-2,3-dihydro-1*H*-3*a*,9*b*-(methanooxymethano)pyrrolo[3,2-*c*]quinoline-4,10(5*H*)-dione, 10a

Colorless solid (92%), Mp: 220–222 °C, IR (KBr): 1709, 1744 cm^−1^; ^1^H NMR (300 MHz, CDCl_3_): *δ* 2.07–2.16 (m, 1H), 2.40–2.58 (m, 1H), 2.61 (s, –NCH_3_, 3H), 2.73 (q, *J* = 9.0, 18.0 Hz, 1H), 3.07–3.14 (m, 1H), 3.81 (s, –OCH_3_, 3H), 5.39 (s, 1H), 6.87–6.90 (m, 2H), 7.02 (d, *J* = 8.1 Hz, 1H), 7.18 (t, *J* = 7.2 Hz, 1H), 7.30 (d, *J* = 8.7 Hz, 2H), 7.39 (t, *J* = 7.2 Hz, 1H), 7.80 (d, *J* = 7.5 Hz, 1H), 9.94 (s, 1H, N–H). ^13^C NMR (75 MHz, CDCl_3_): 26.7, 34.3, 52.4, 55.2, 59.1, 73.0, 79.4, 113.7, 114.6, 116.5, 123.8, 126.0, 127.5, 129.9, 130.7, 136.7, 159.8, 170.2, 173.7 ppm. Mass: *m*/*z* 364.14 (M^+^). Anal. calcd. For C_21_H_20_N_2_O_4_: C, 69.22, H, 5.53, N, 7.69%; found: C, 69.27, H, 5.60, N, 7.63%.

#### (3aR*,9bR*,12R*)-1-Methyl-12-(*o*-tolyl)-2,3-dihydro-1*H*-3*a*,9*b*(methanooxymethano)pyrrolo[3,2-*c*]quinoline-4,10(5*H*)-dione, 10b

Colorless solid (93%), Mp: 268–270 °C, IR (KBr): 1709, 1744 cm^−1^; ^1^H NMR (300 MHz, CDCl_3_): *δ* 2.19 (s, –NCH_3_, 3H), 2.37–2.52 (m, 2H), 2.59 (s, 3H), 2.80 (q, *J* = 9.1, 17.7 Hz, 1H), 3.07–3.15 (m, 1H), 5.68 (s, 1H), 6.96 (d, *J* = 7.8 Hz, 1H), 7.13–7.29 (m, 4H) 7.41 (t, *J* = 7.8 Hz, 1H), 7.47–7.50 (m, 1H), 7.80 (d, *J* = 7.5 Hz, 1H), 9.39 (s, 1H, N–H). ^13^C NMR (75 MHz, CDCl_3_): 19.2, 26.0, 34.4, 52.5, 59.0, 73.6, 76.4, 114.7, 116.3, 123.9, 125.8, 127.1, 128.8, 130.1, 130.7, 131.0, 131.6, 135.8, 136.8, 169.7, 173.8 ppm. Mass: *m*/*z* 348.14 (M^+^). Anal. calcd. For C_21_H_20_N_2_O_3_: C, 72.40; H, 5.79; N, 8.04%; found: C, 72.45, H, 6.84, N, 7.99%.

#### (3aR*,9bR*,12R*)-1-Methyl-12-(4-nitrophenyl)-2,3-dihydro-1*H*-3*a*,9*b*-(methanooxymethano)pyrrolo[3,2-*c*]quinoline-4,10(5*H*)-dione, 10c

Colorless solid (87%), Mp: 250–252 °C, IR (KBr): 1720, 1745 cm^−1^; ^1^H NMR (300 MHz, CDCl_3_): *δ* 1.90–2.04 (m, 1H), 2.44–2.55 (m, 1H), 2.62 (s, –NCH_3_, 3H), 2.76 (q, *J* = 9.0, 17.7 Hz, 1H), 3.09–3.16 (m, 1H), 5.49 (s, 1H), 7.02 (d, *J* = 8.1 Hz, 1H), 7.24 (t, *J* = 7.5 Hz, 1H), 7.46 (t, *J* = 7.8 Hz, 1H), 7.61–7.64 (m, 2H), 7.79 (d, *J* = 7.8 Hz, 1H), 8.23–8.23 (m, 2H), 9.87 (s, 1H, N–H). ^13^C NMR (75 MHz, CDCl_3_): 26.9., 34.1, 52.2, 58.6, 73.0, 78.2, 114.2, 116.5, 123.5, 124.2, 127.3, 129.9, 131.1, 136.4, 141.3, 148.1, 169.6, 172.7 ppm. Mass: *m*/*z* 379.11 (M^+^): anal. calcd. For C_20_H_17_N_3_O_5_: C, 63.32, H, 4.52, N, 11.08%; found: C, 63.37, H, 4.55, N, 11.10%.

#### (6aR*,7aR*,11aR*,14R*)-14-(Pyren-1-yl)-7*a*,8,9,10-tetrahydro-5*H*-6*a*,11*a*-(methanooxymethano)pyrrolizino[2,3-*c*]quinoline-6,12(7*H*)-dione, 12a

Colorless solid (94%), Mp: 248–250 °C, IR (KBr): 1719, 1748 cm^−1^; ^1^H NMR (300 MHz, CDCl_3_ + DMSO-d_6_): *δ* 1.32–1.34 (m, 1H), 1.60–1.68 (m, 2H), 1.99–2.04 (m, 2H), 2.39–2.44 (m, 1H), 2.53–2.63 (m, 2H), 3.66–3.72 (m, 1H), 6.59 (s, 1H), 7.18–7.48 (m, 3H), 7.78 (d, *J* = 8.1 Hz, 1H), 8.05–8.32 (m, 9H), 11.17 (s, 1H, N–H). ^13^C NMR (75 MHz, CDCl_3_ + DMSO-d_6_): 29.9, 36.9, 39.5, 53.9, 64.3, 68.1, 81.9, 119.6, 121.5, 127.6, 128.5, 128.9, 129.2, 129.3, 129.5, 130.3, 130.7, 131.3, 132.2, 132.5, 132.7, 133.0, 133.3, 133.6, 135.1, 135.6, 135.9, 136.1, 143.8, 173.2, 180.5 ppm. Mass: *m*/*z* 484.19 (M^+^). Anal. calcd. For C_32_H_24_N_2_O_3_: C, 79.32, H, 4.99, N, 5.78% found: C, 79.35, H, 4.96, N, 5.75%.

#### (6aR*,7aR*,11aR*,14R*)-2-Chloro-14-(pyren-1-yl)-7*a*,8,9,10-tetrahydro-5*H*-6*a*,11*a*-(methanooxymethano)pyrrolizino[2,3-*c*]quinoline-6,12(7*H*)-dione, 12b

Colorless solid (91%), Mp: 256–258 °C, IR (KBr): 1719, 1748 cm^−1^; ^1^H NMR (300 MHz, CDCl_3_ + DMSO-d_6_): *δ* 1.32–1.36 (m, 1H), 1.67–1.71 (m, 2H), 1.96–2.11 (m, 2H), 2.42–2.68 (m, 3H), 3.67–3.72 (m, 1H), 6.65 (s, 1H), 7.27–8.34 (m, 12H), 11.29 (s, 1H, N–H). ^13^C NMR (75 MHz, CDCl_3_ + DMSO-d_6_): 24.5, 31.4, 34.6, 48.6, 58.8, 62.9 76.4, 116.0, 117.9, 122.3, 123.7, 123.9, 124.1, 124.2, 125.1, 125.5, 126.0, 126.5, 127.3, 127.5, 127.8, 127.9, 128.1, 128.4, 129.9, 130.4, 130.6, 131.0, 137.3, 167.8, 174.9 ppm. Mass: *m*/*z* 518.19 (M^+^). Anal. calcd. For C_32_H_23_ClN_2_O_3_: C, 74.06, H, 4.47, N, 5.40% found: C, 74.09, H, 4.45, N, 5.43%.

#### (6aR*,7aR*,11aR*,14R*)-2-Bromo-14-(pyren-1-yl)-7*a*,8,9,10-tetrahydro-5*H*-6*a*,11*a*-(methanooxymethano)pyrrolizino[2,3-*c*]quinoline-6,12(7*H*)-dione, 12c

Colorless solid (92%), Mp: 259–261 °C, IR (KBr): 1725, 1756 cm^−1^; ^1^H NMR (300 MHz, CDCl_3_ + DMSO-d_6_): *δ* 1.34–1.42 (m, 1H), 1.68–1.80 (m, 2H), 2.04–2.17 (m, 3H), 2.45–2.57 (m, 1H), 2.73–2.80 (m, 1H), 3.73–3.79 (m, 1H), 6.56 (s, 1H), 7.23 (s, 1H), 7.54 (s, 1H), 7.66 (s, 1H), 8.00–8.26 (m, 9H), 11.16 (s, 1H, N–H). ^13^C NMR (75 MHz, CDCl_3_ + DMSO-d_6_): 29.8, 36.7, 39.6, 54.0, 64.2, 68.2, 82.3, 121.4, 123.2, 127.1, 129.1, 129.3, 130.1, 130.6, 131.0, 132.0, 132.5, 132.9, 133.3, 135.1, 135.9, 136.3, 138.5, 173.2, 180.3 ppm. Mass: *m*/*z* 562.11 (M^+^). Anal. calcd. For C_32_H_23_BrN_2_O_3_: C, 68.21, H, 4.11, N, 4.97% found: C, 68.24, H, 4.08, N, 4.95%

## Conflicts of interest

There is no conflict of interest.

## Supplementary Material

RA-012-D2RA02851D-s001

RA-012-D2RA02851D-s002
