# Supplementary material for: Environmentally friendly domino multicomponent strategy for the synthesis of pyrroloquinolinone hybrid heterocycles
Source: RSC Adv. 2022 May 25;12(24):15440–6. doi: 10.1039/d2ra02851d (PMC9131013; doi:10.1039/d2ra02851d)
Supplement: RA-012-D2RA02851D-s001 [file RA-012-D2RA02851D-s001.pdf]

Electronic Supplementary Material (ESI) for RSC Advances.
This journal is © The Royal Society of Chemistry 2022

Supplementary data

Environmental friendly domino multicomponent strategy for the synthesis of pyrroloquinolinone hybrid heterocycles

Suresh Mani,[a] Rajesh Raju,[a*] Raghavachary Raghunathan,[a*] Natarajan Arumugam,[b] Abudulrahman I. Almansour,[b] Raju Suresh Kumar,[b] Karthikeyan Perumal[c]

[a]*Department of Organic Chemistry, University of Madras, Guindy Campus, Chennai-600 025*

[b]*Department of Chemistry, College of Science, King Saud University, P.O. Box 2455, Riyadh, 11451, Saudi Arabia*

[c]*Department of Chemistry and Biochemistry, The Ohio State University, 151 W. Woodruff Ave, Columbus, OH 43210, USA*

Selected ^{1}H and ^{13}C NMR spectrums

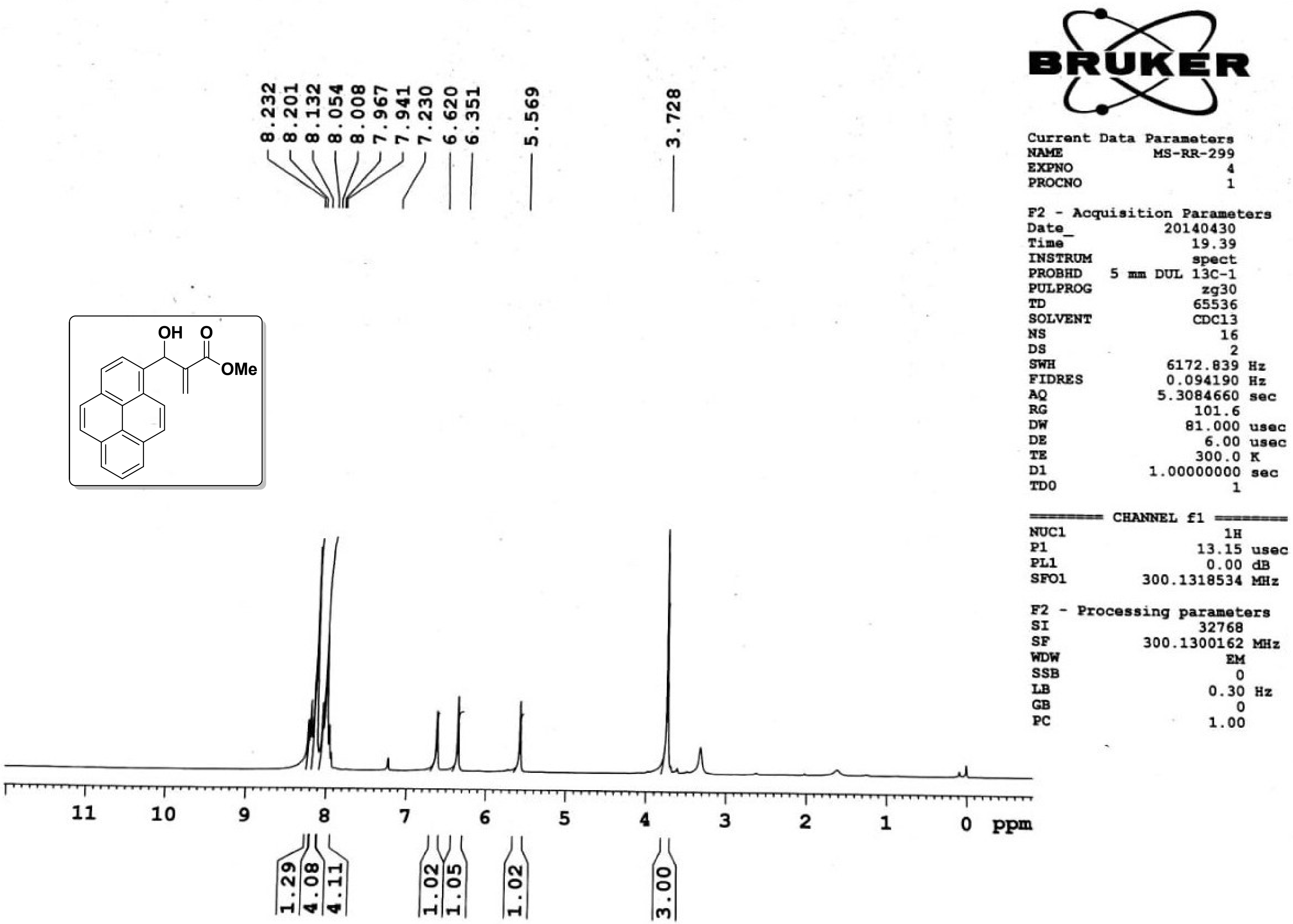

^{1}H-NMR spectrum of compound **3**

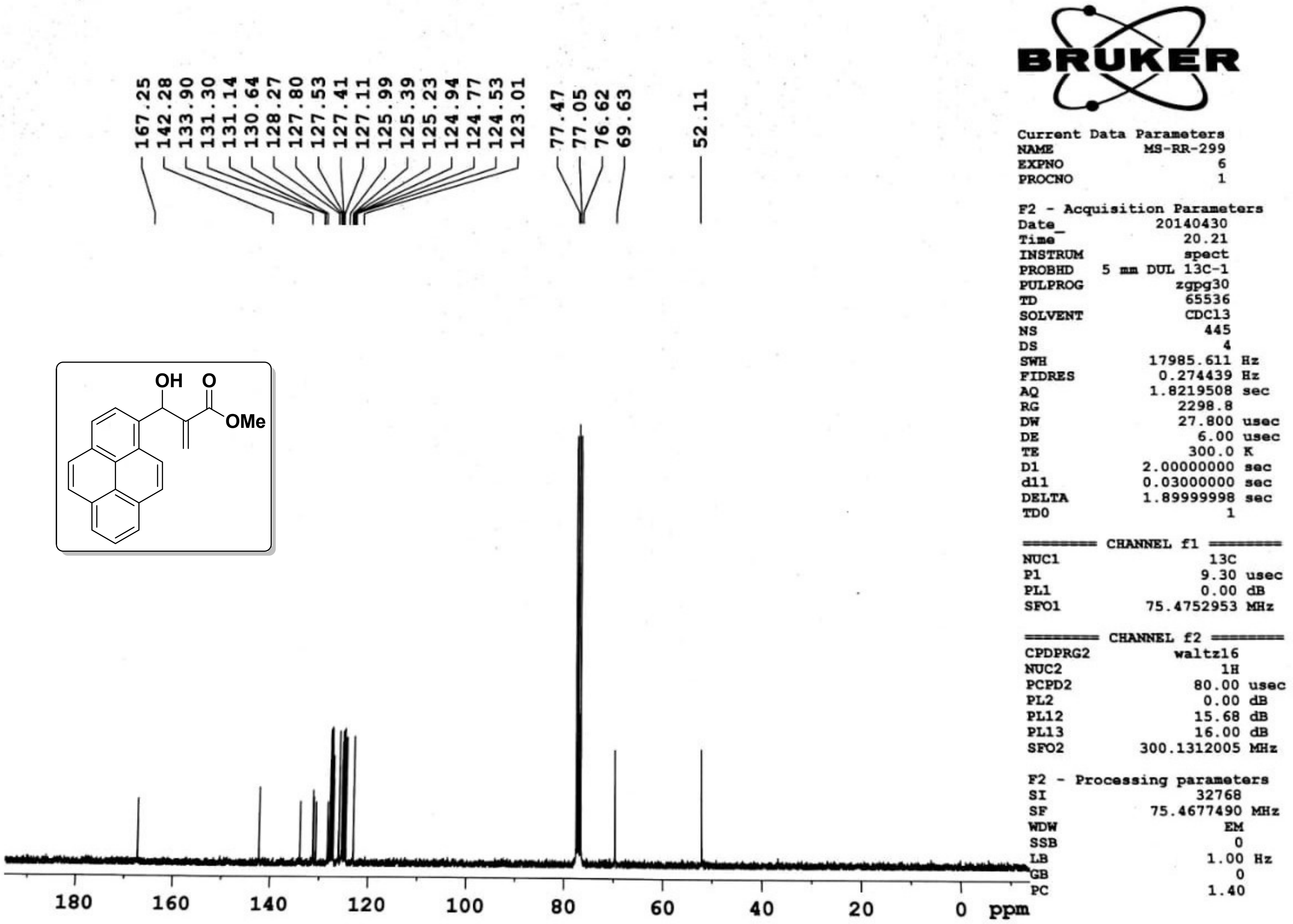

^{13}C-NMR spectrum of compound **3**

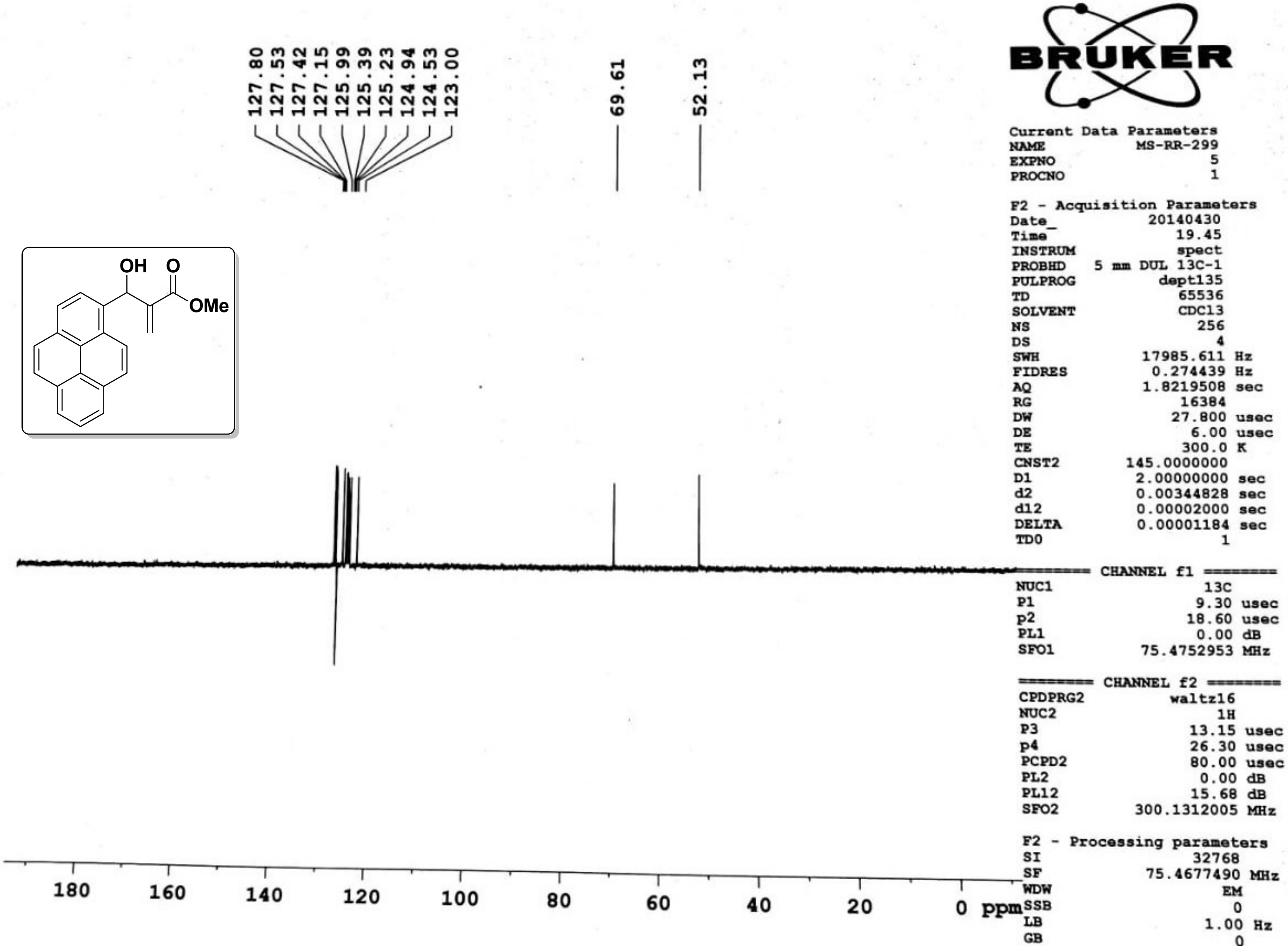

DEPT-NMR spectrum of compound **3**

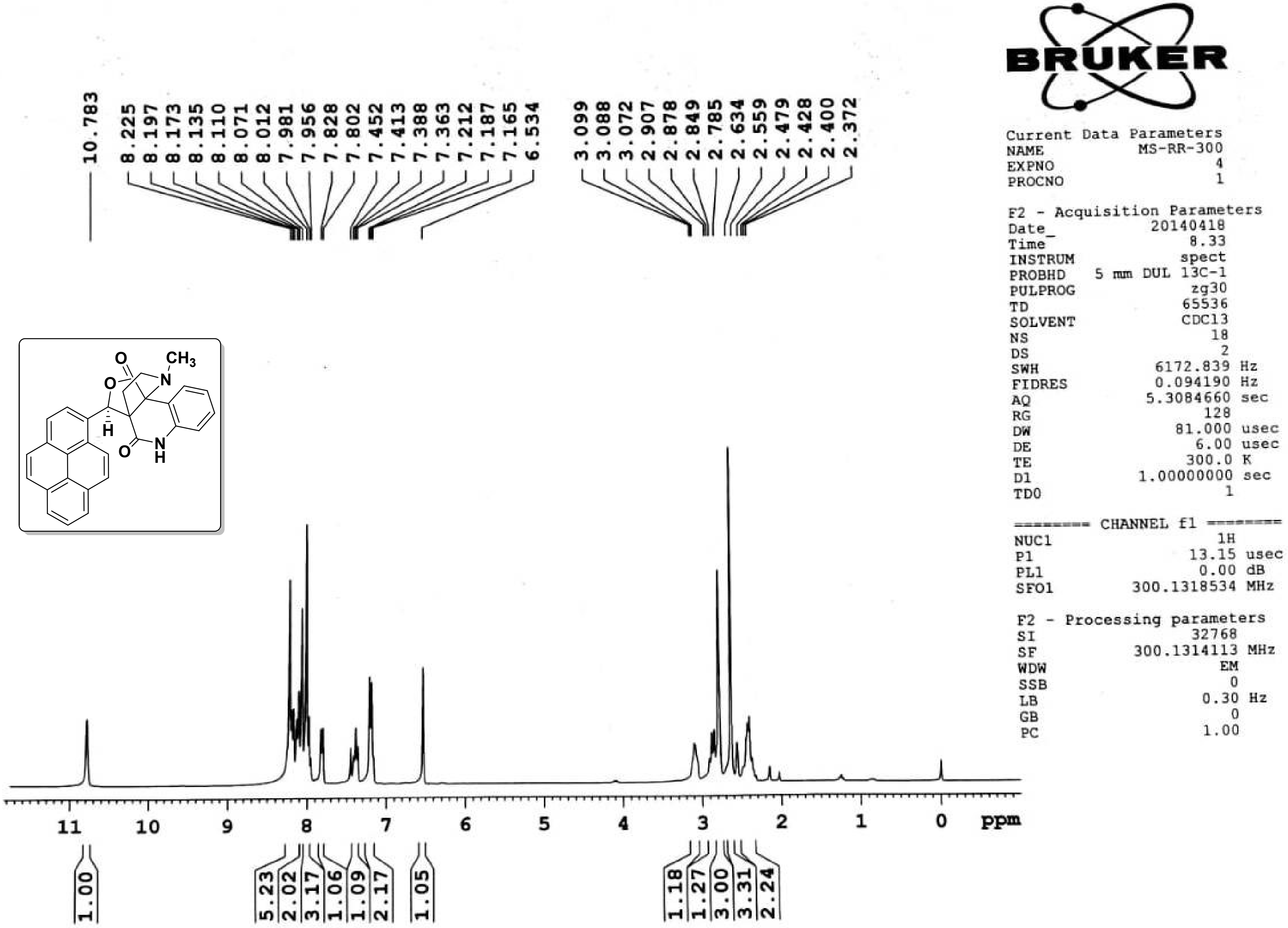

^{1}H NMR spectrum of compound **9a**

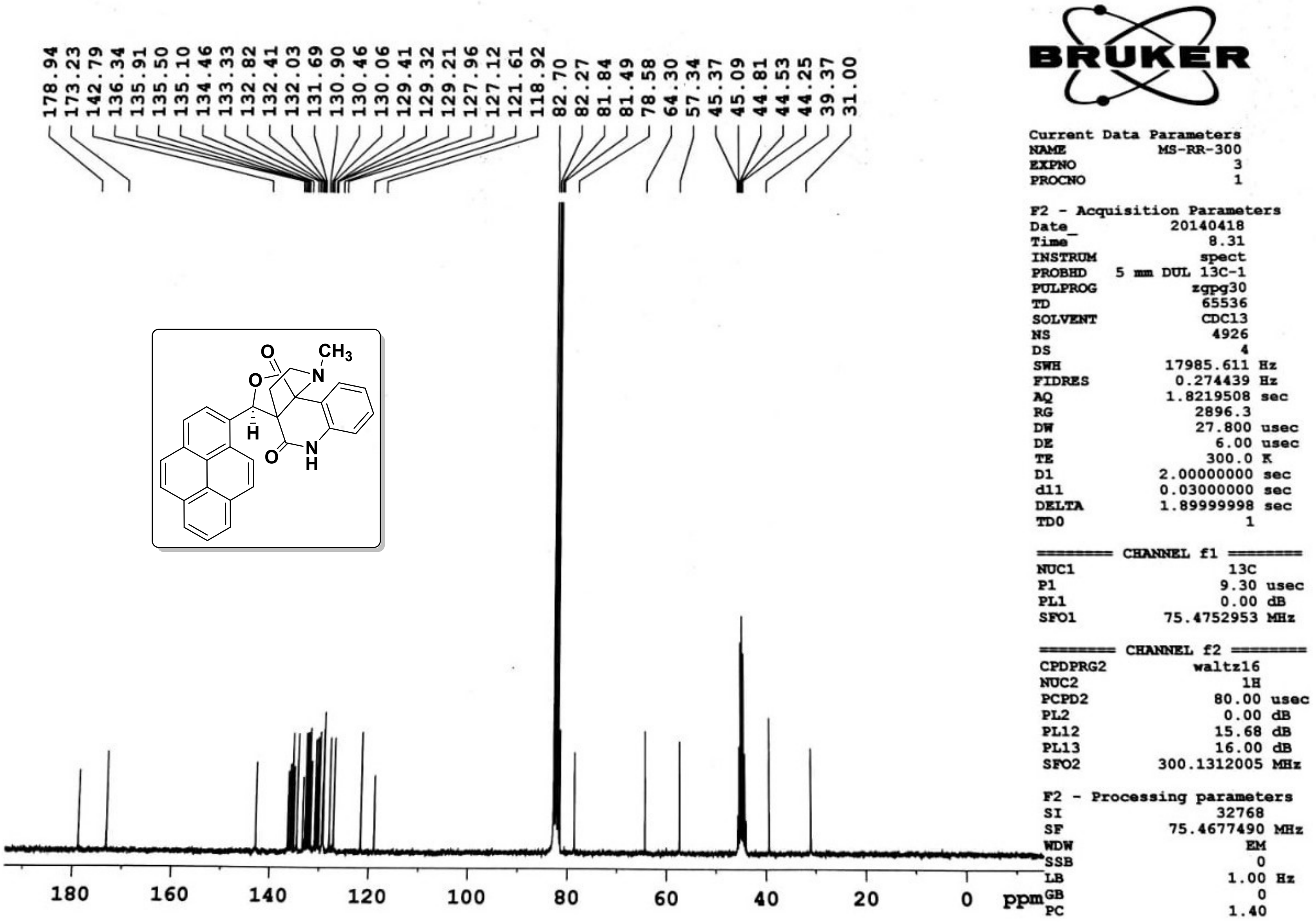

^{13}C NMR spectrum of compound **9a**

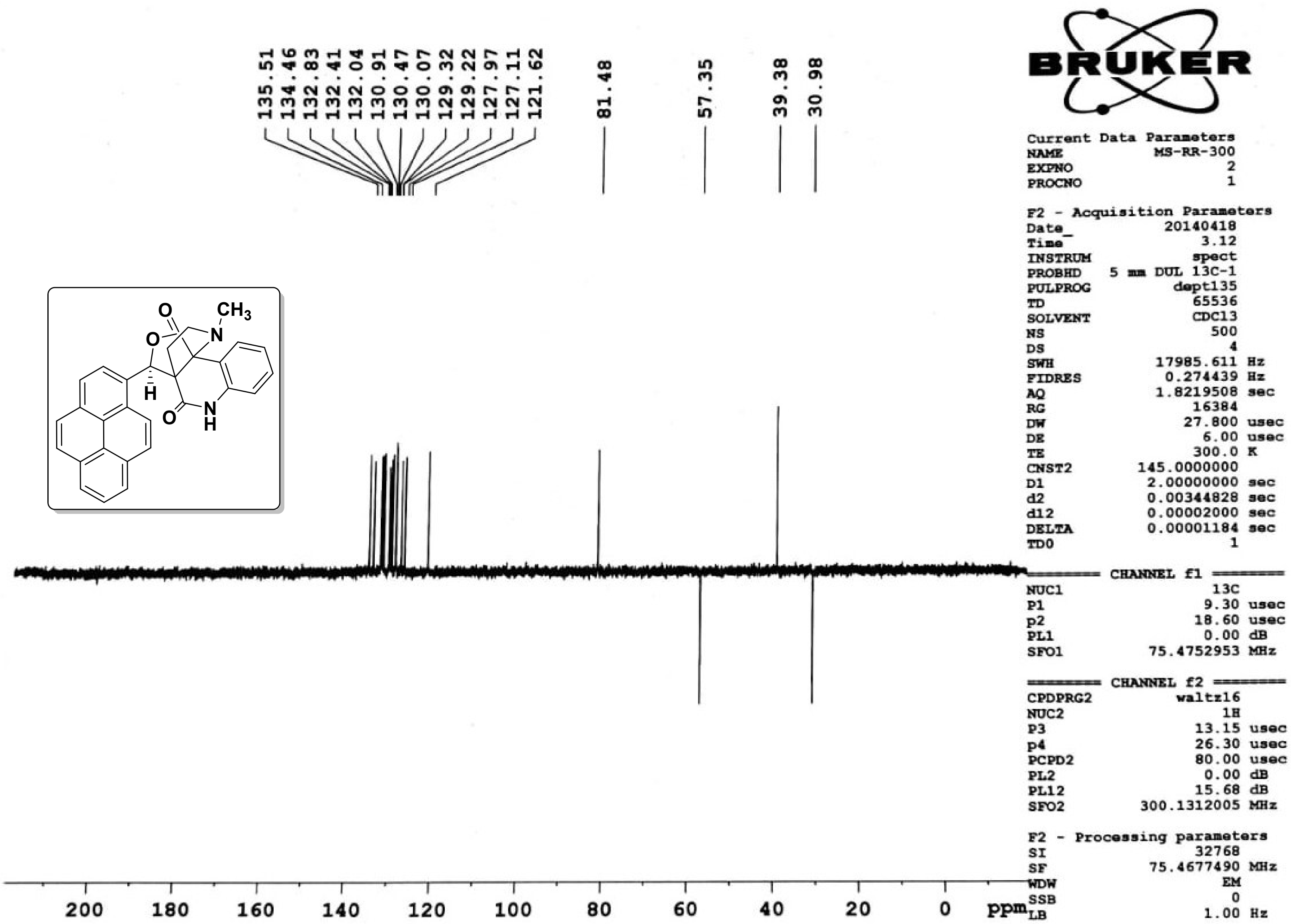

DEPT 135 NMR spectrum of compound **9a**

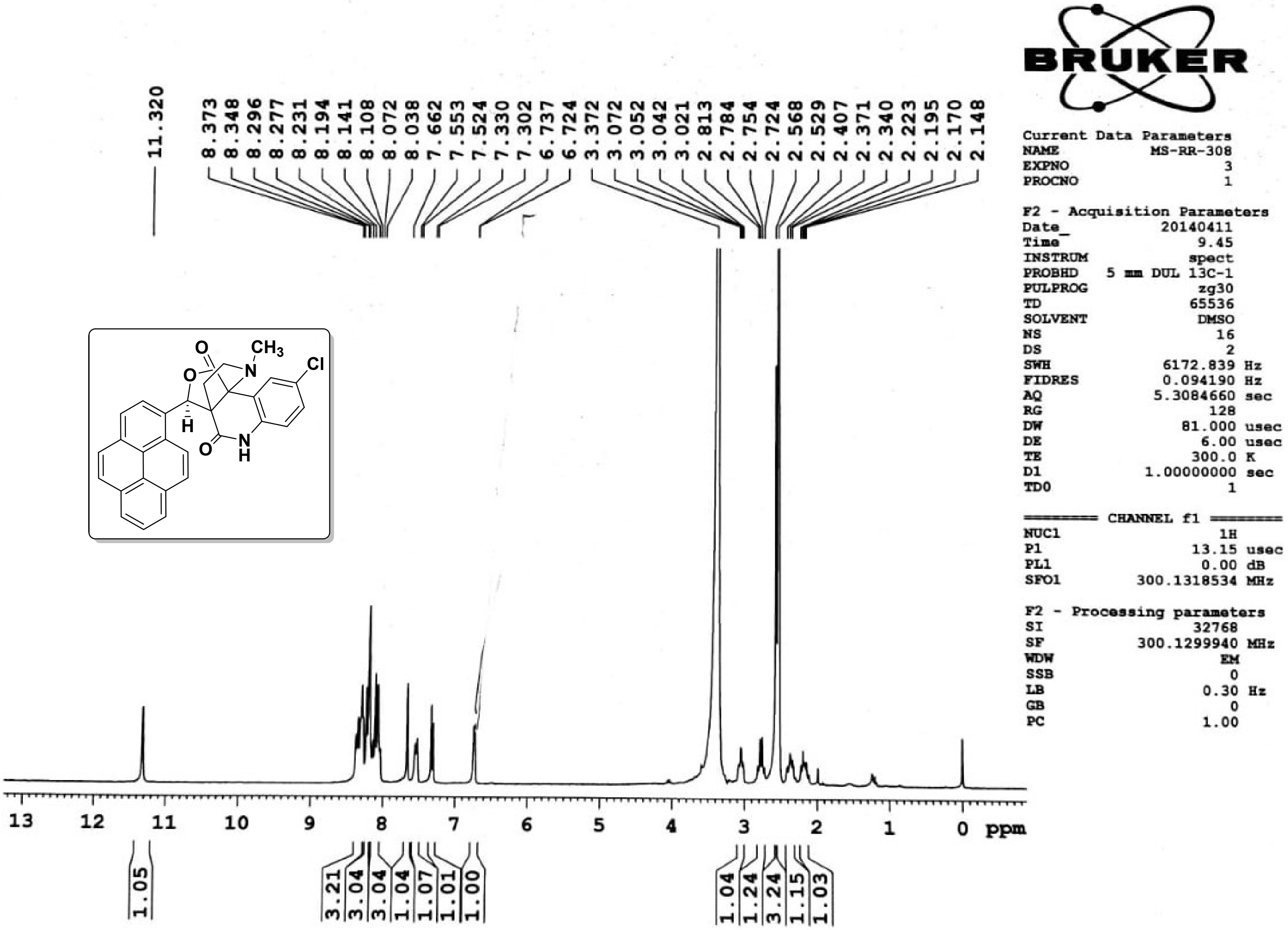

^{1}H NMR spectrum of compound **9b**

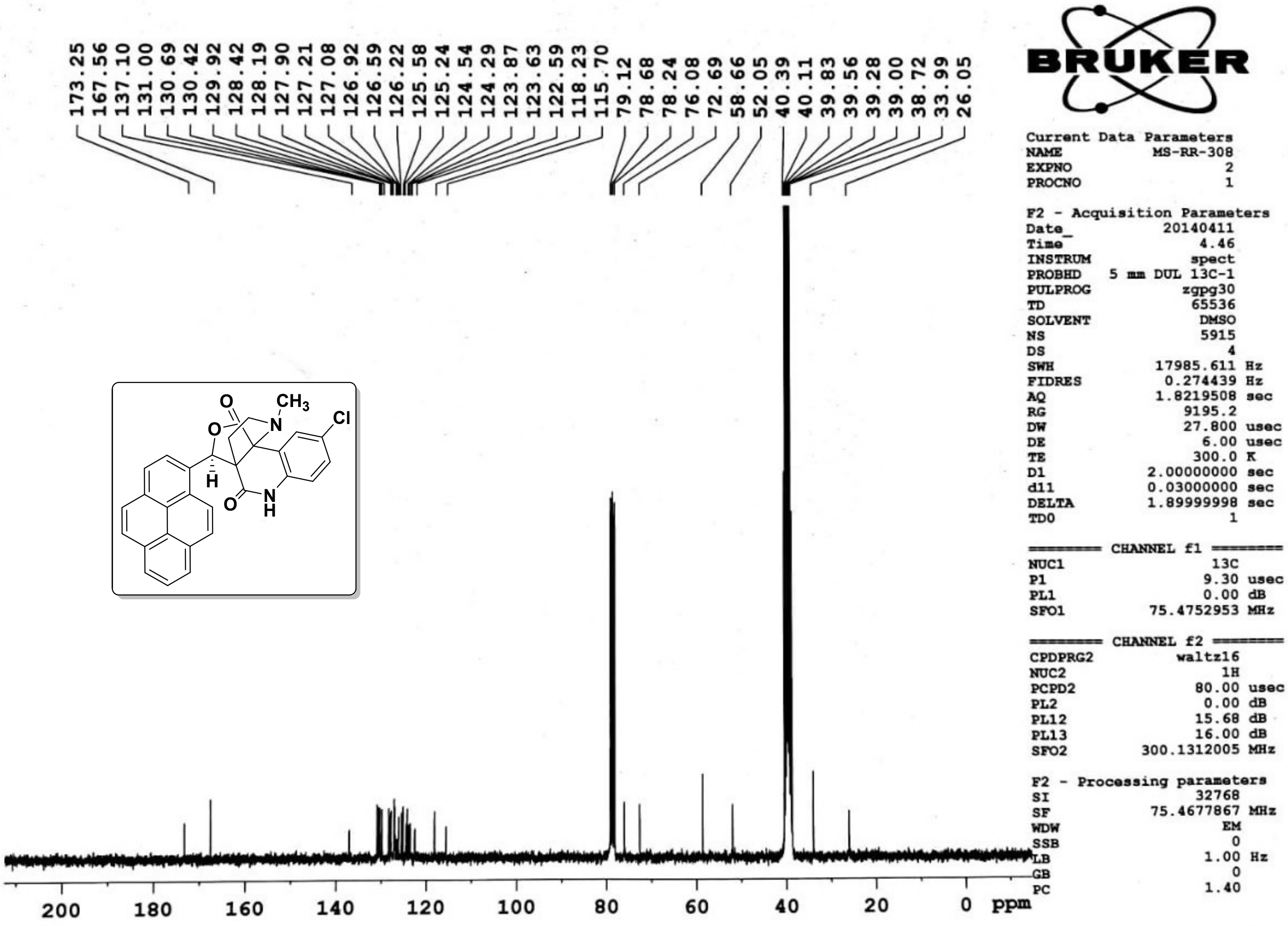

^{13}C NMR spectrum of compound **9b**

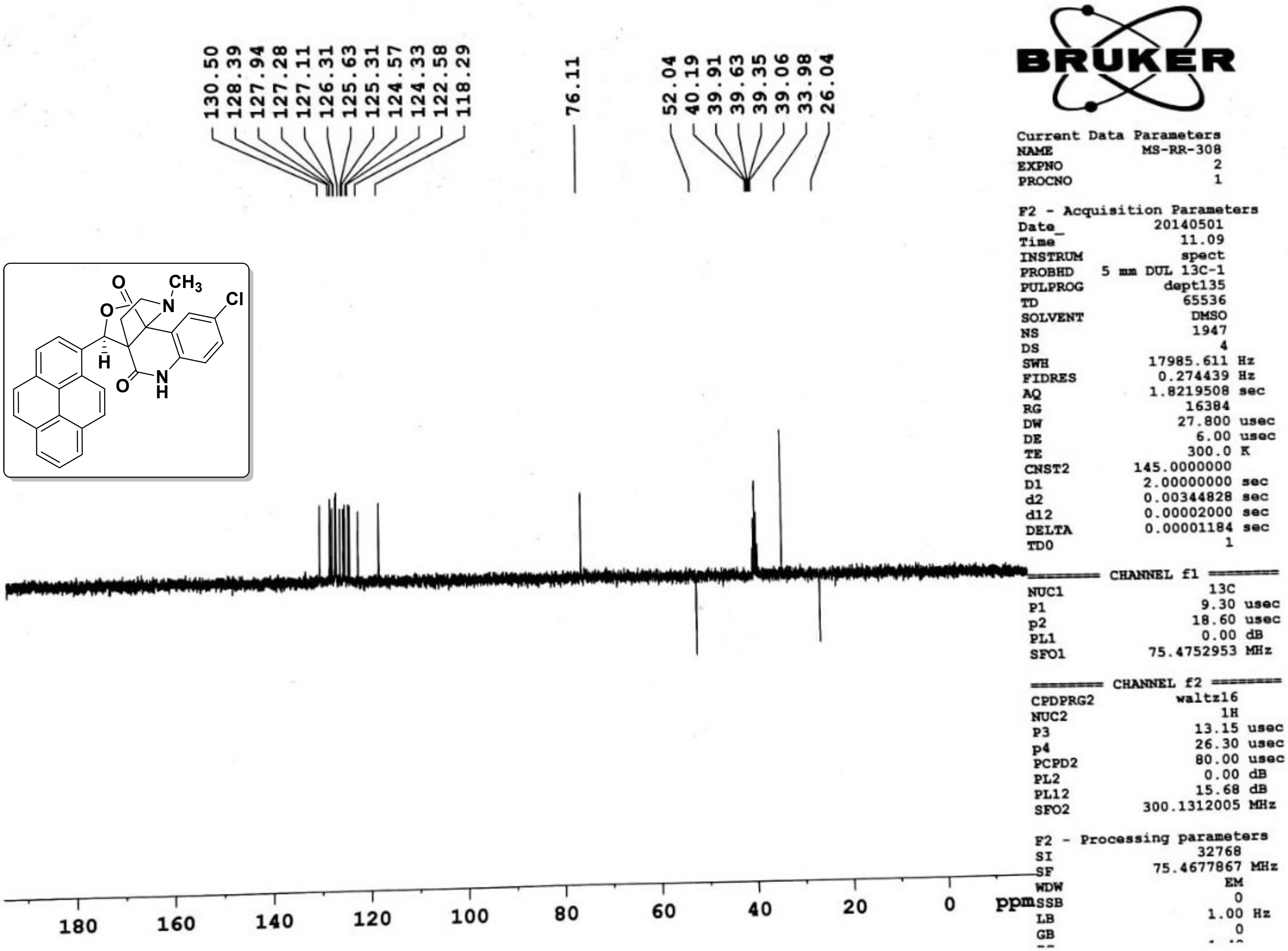

DEPT 135 NMR spectrum of compound **9b**

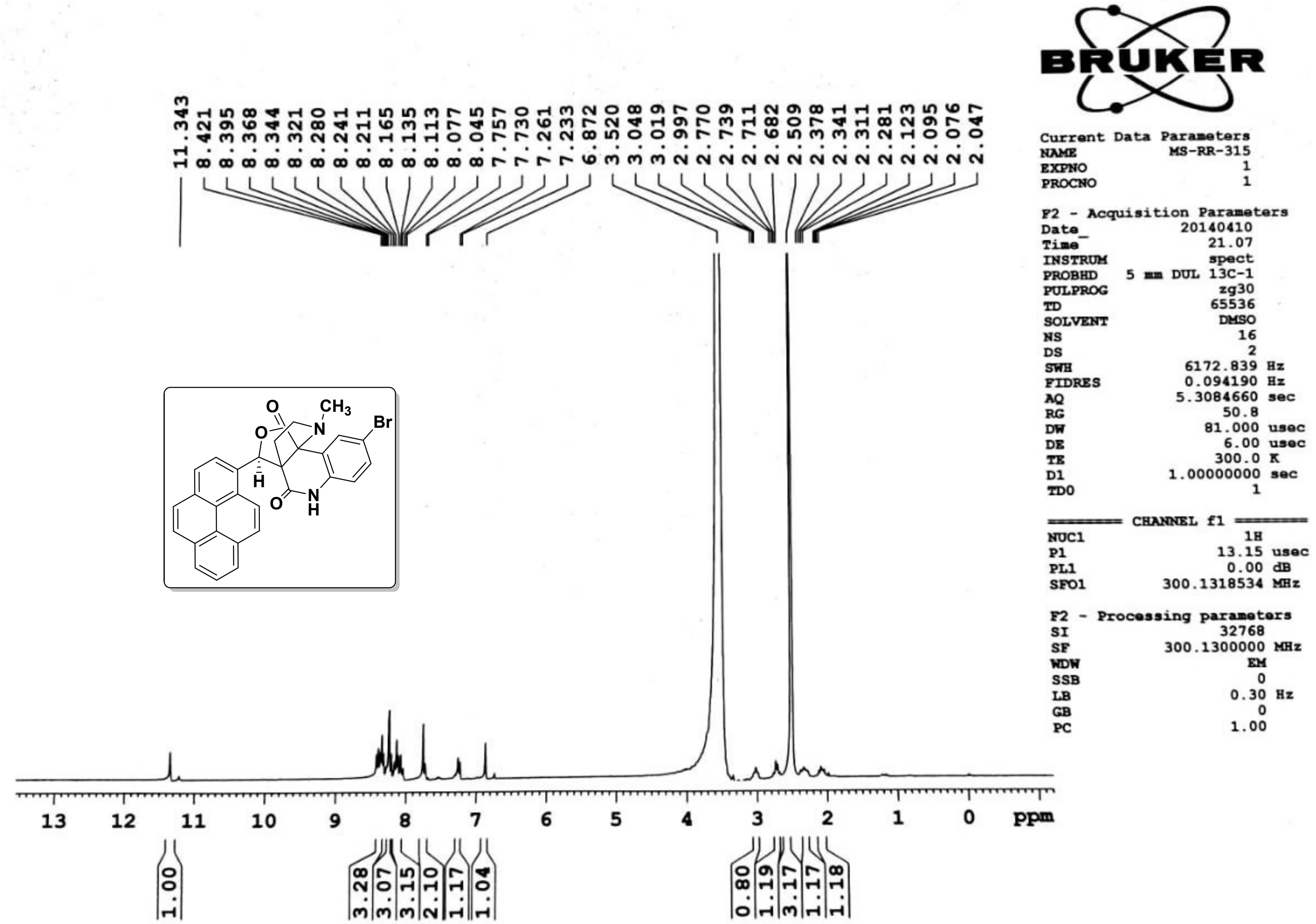

^{1}H NMR spectrum of compound **9c**

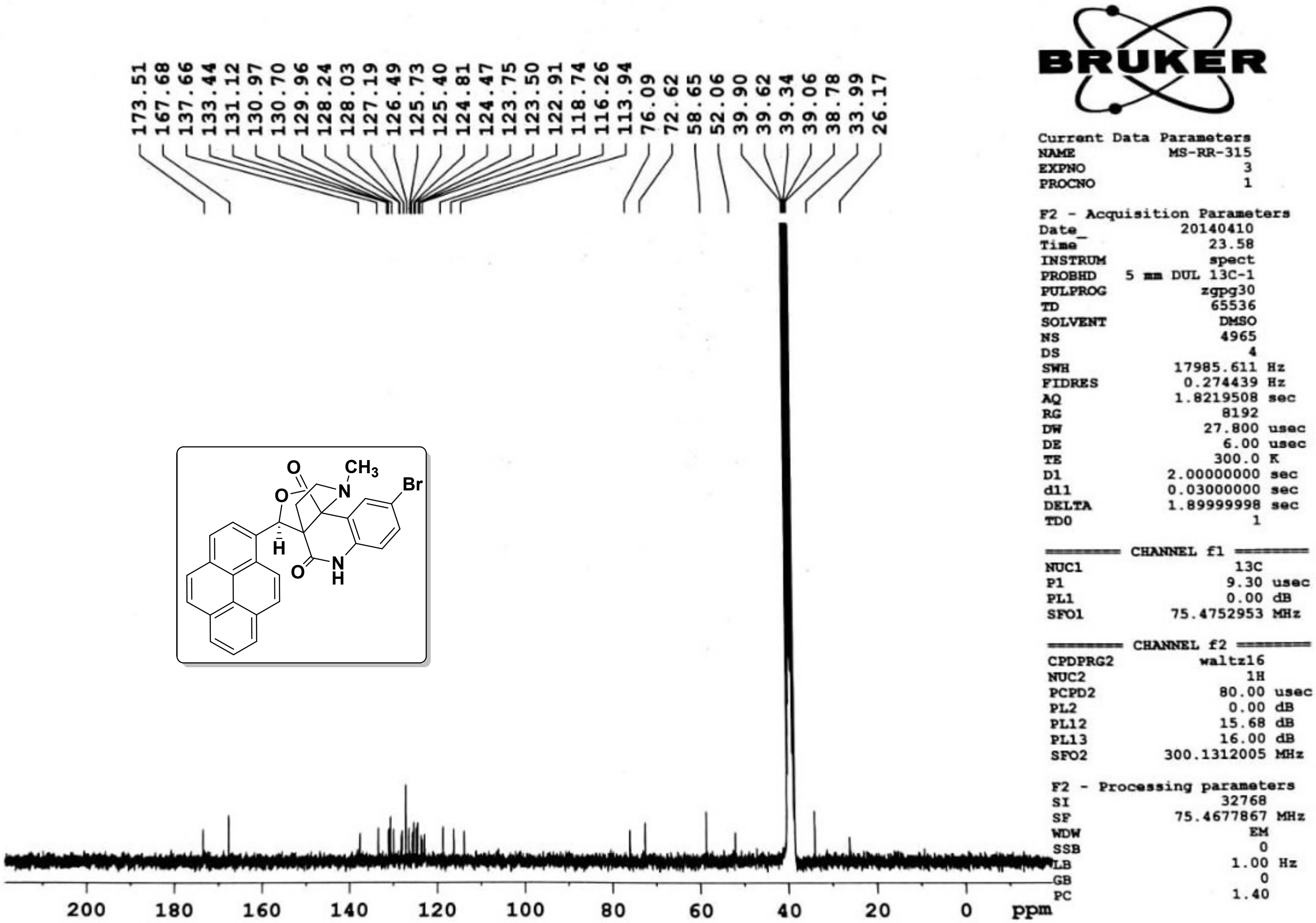

^{13}C NMR spectrum of compound **9c**

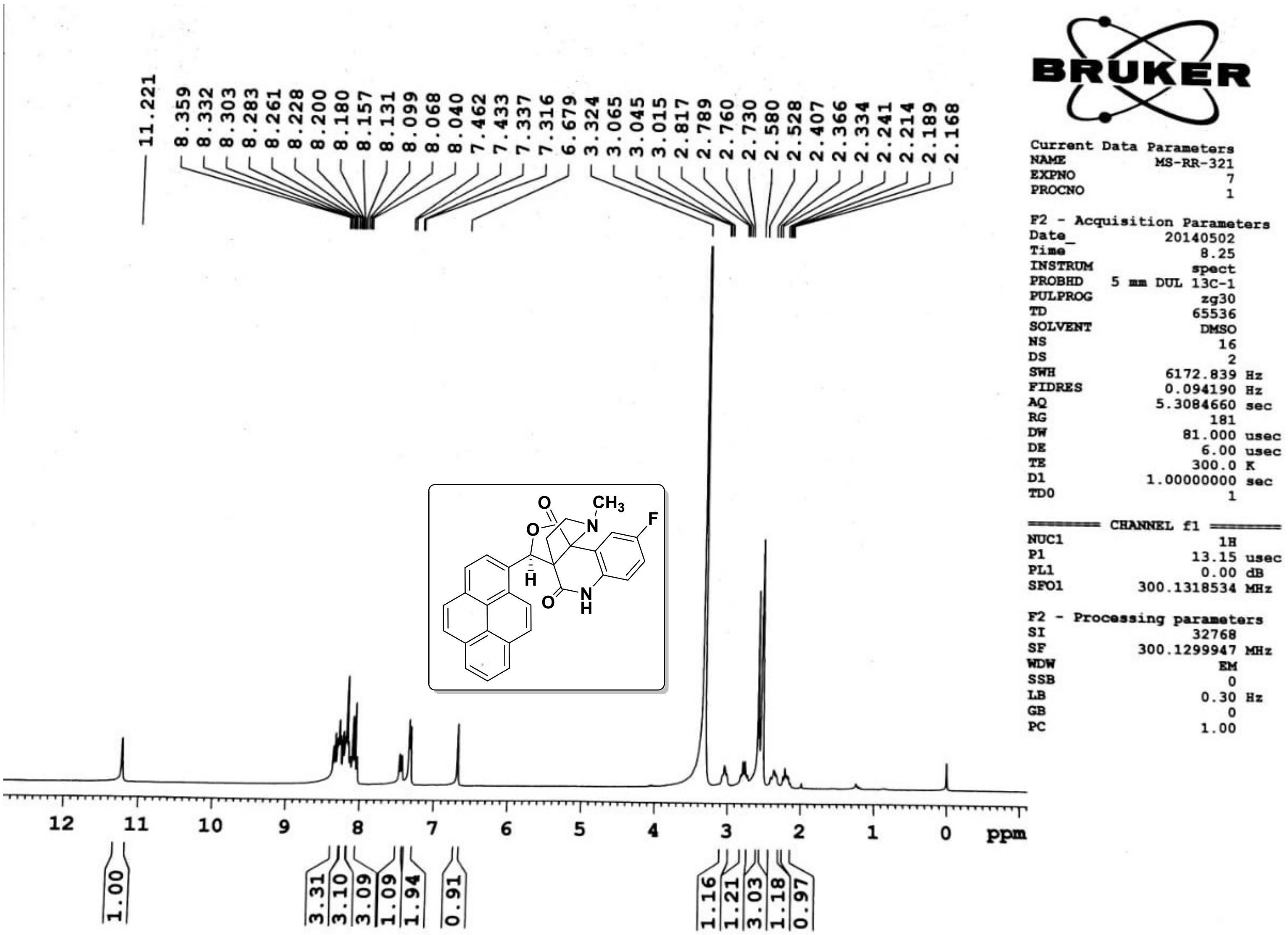

^{1}H NMR spectrum of compound **9d**

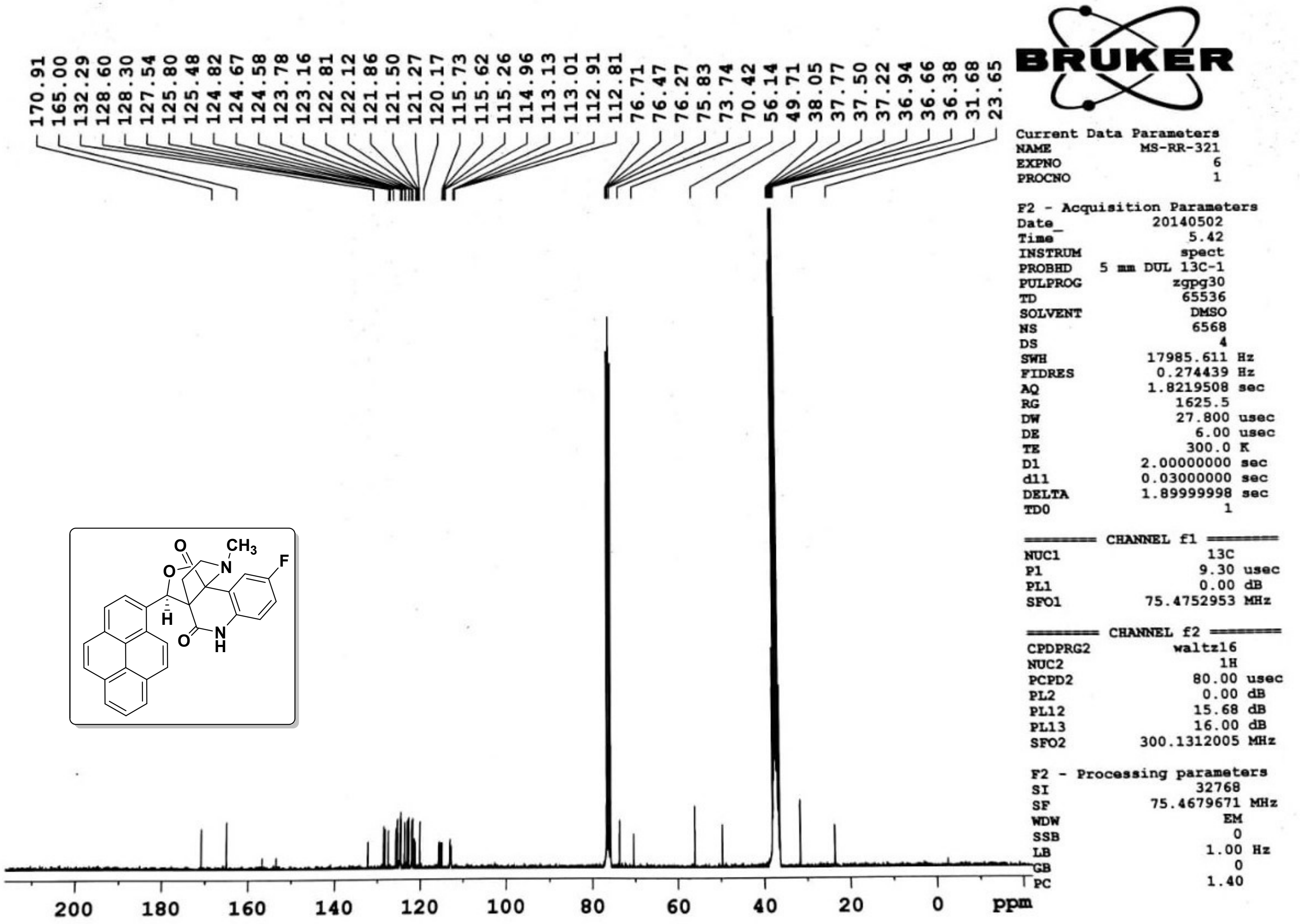

^{13}C NMR spectrum of compound **9d**

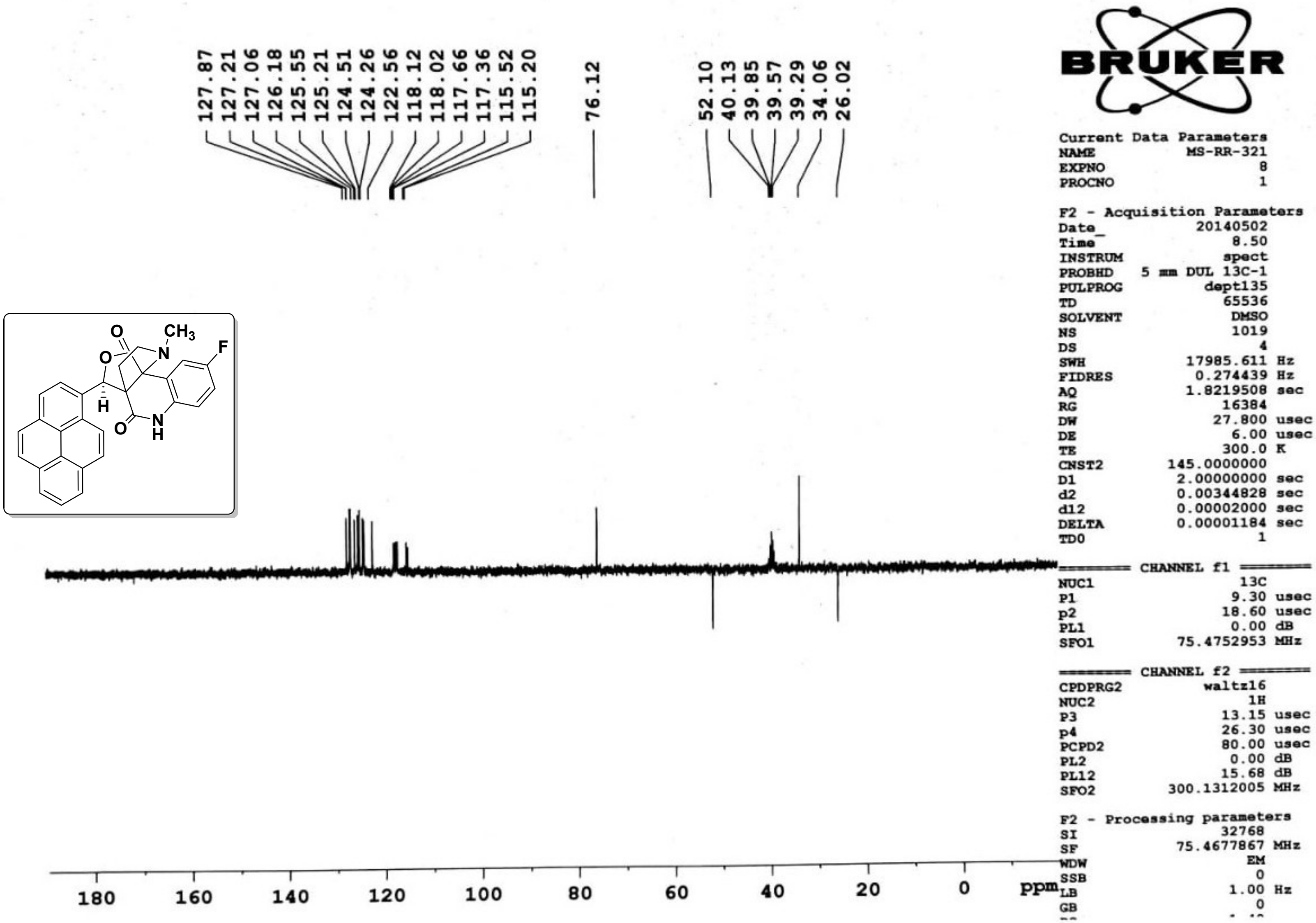

DEPT 135 NMR spectrum of compound **9d**

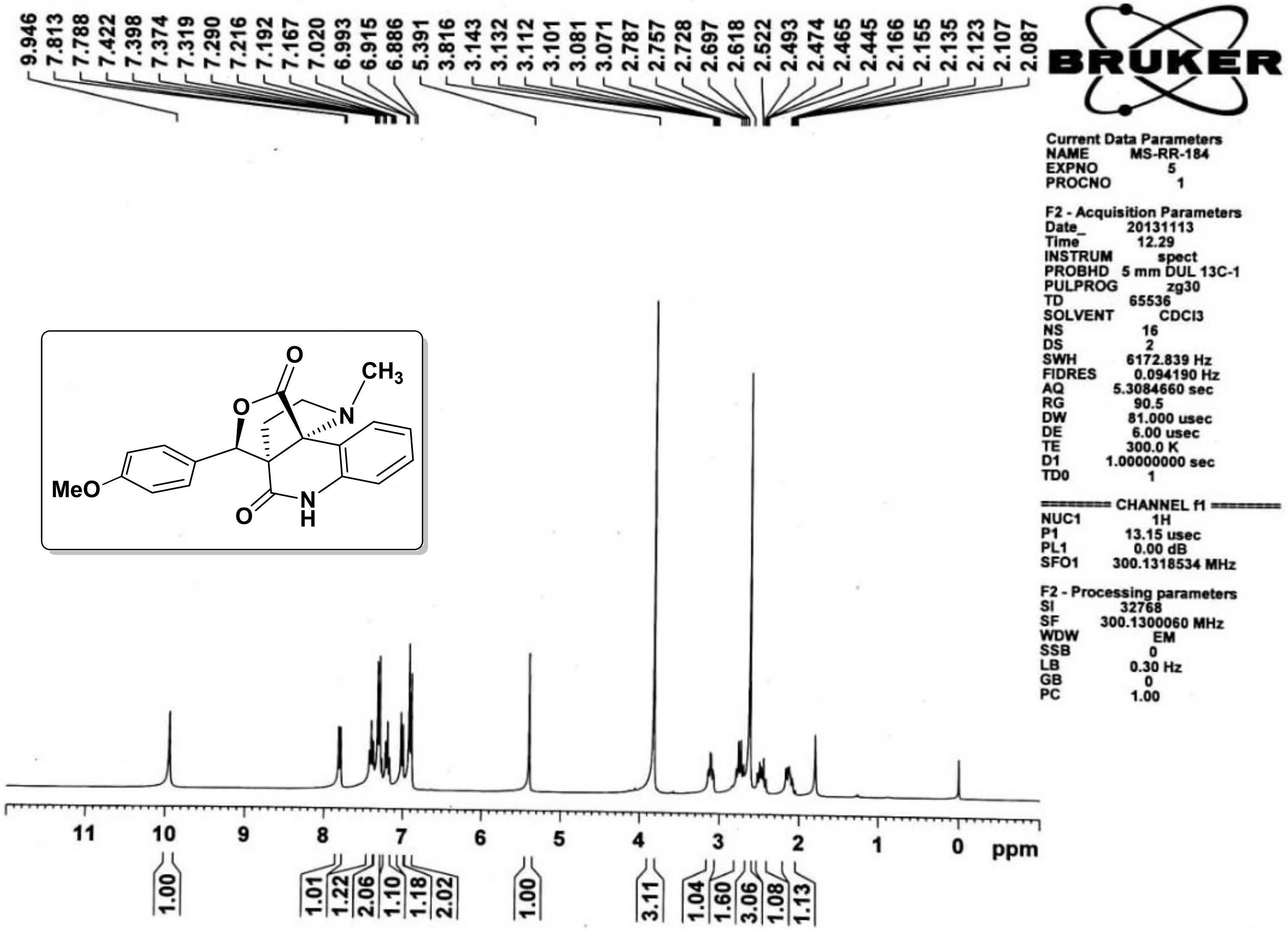

^{1}H-NMR spectrum of compound **10a**

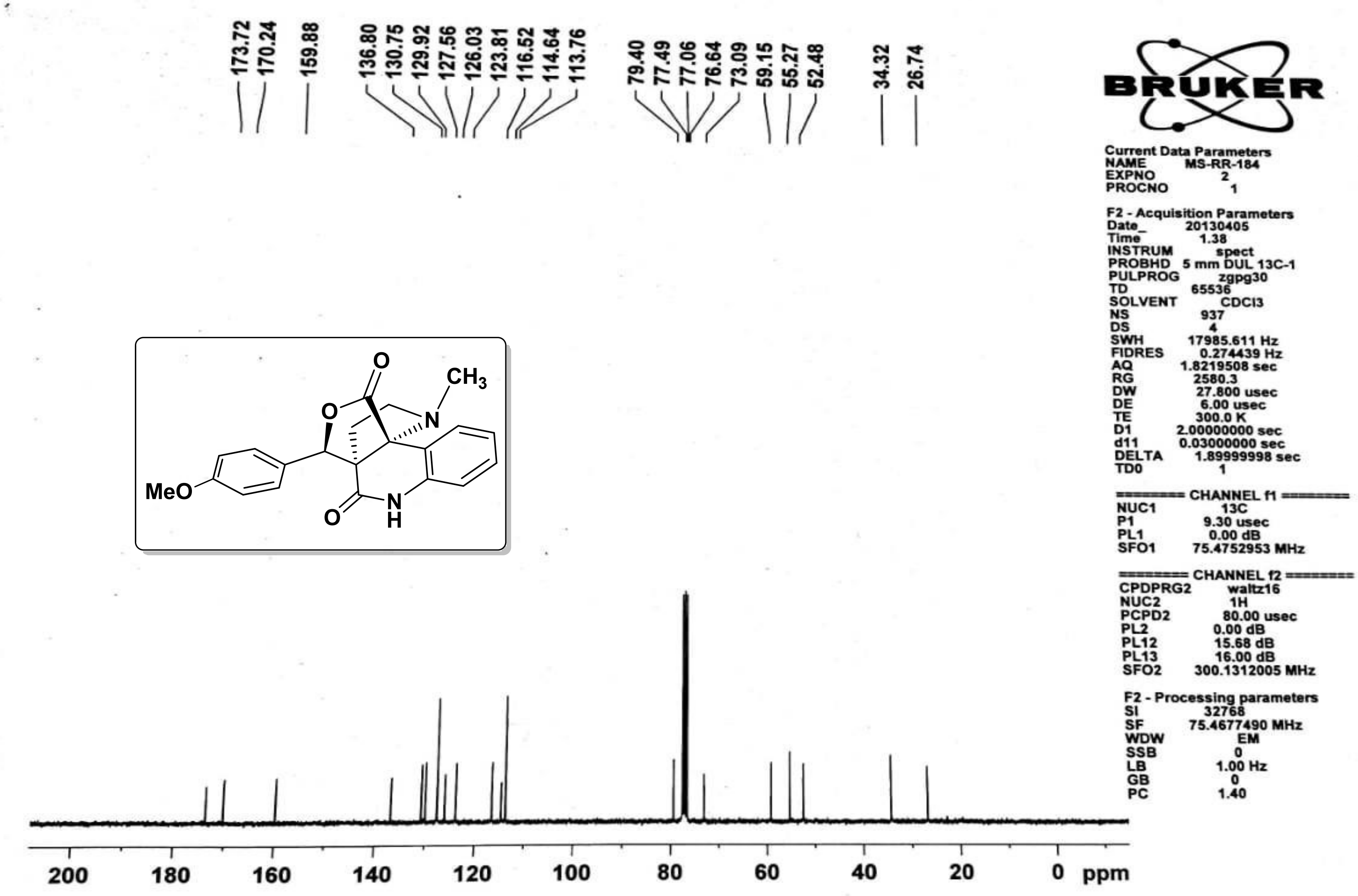

^{13}C-NMR spectrum of compound **10a**

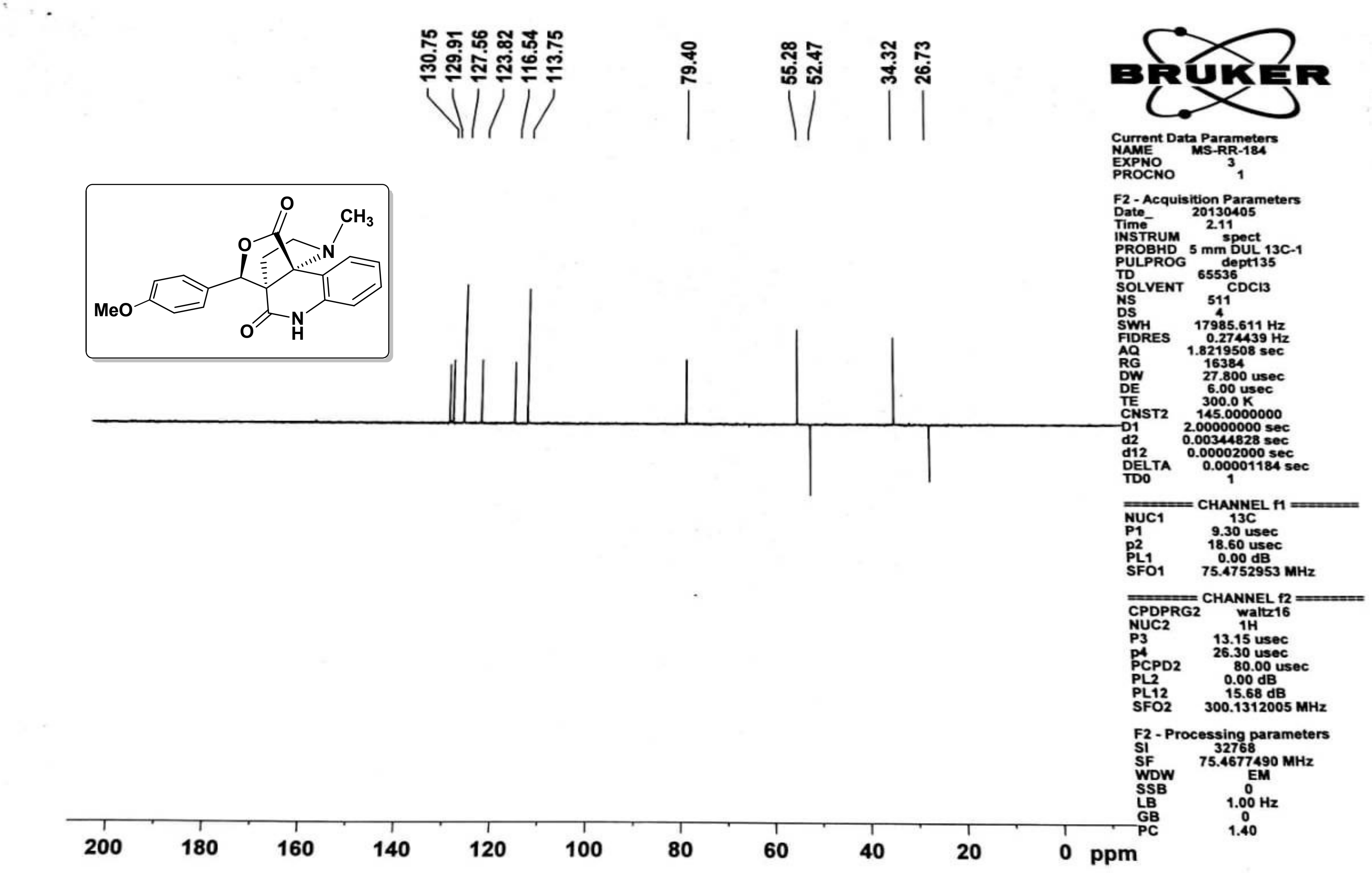

DEPT 135-NMR spectrum of compound **10a**

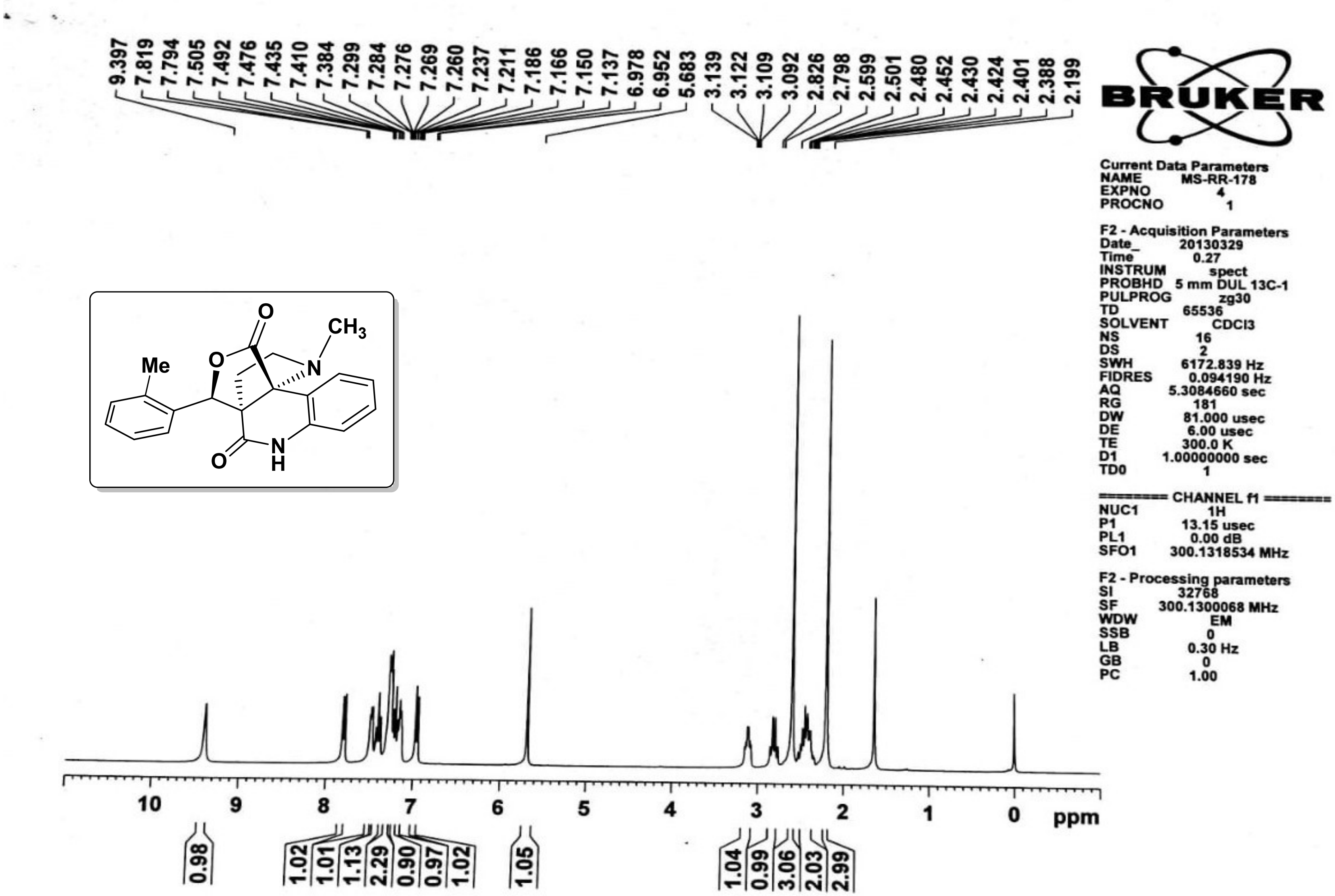

^{1}H NMR spectrum of compound **10b**

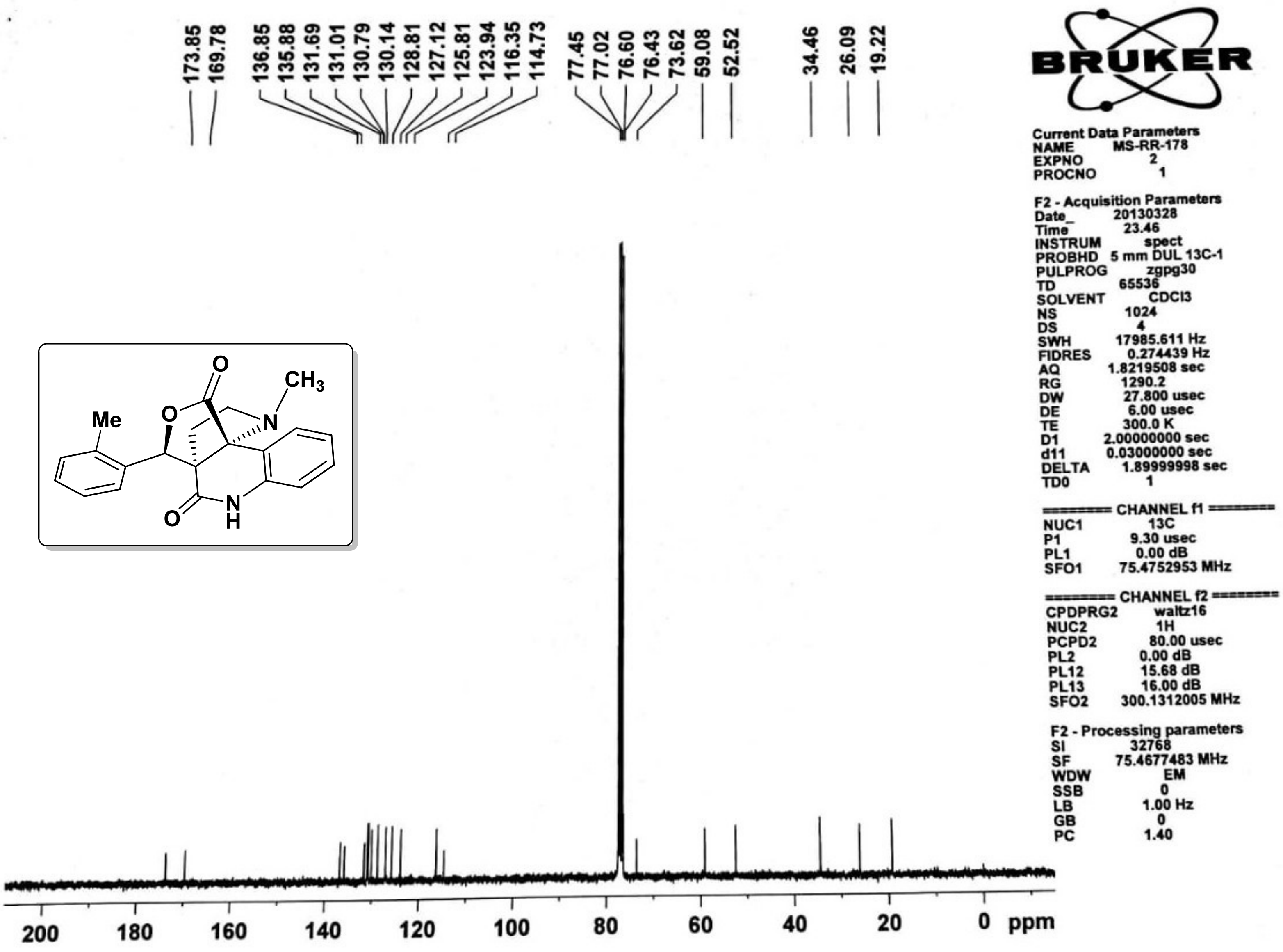

^{13}C-NMR spectrum of compound **10b**

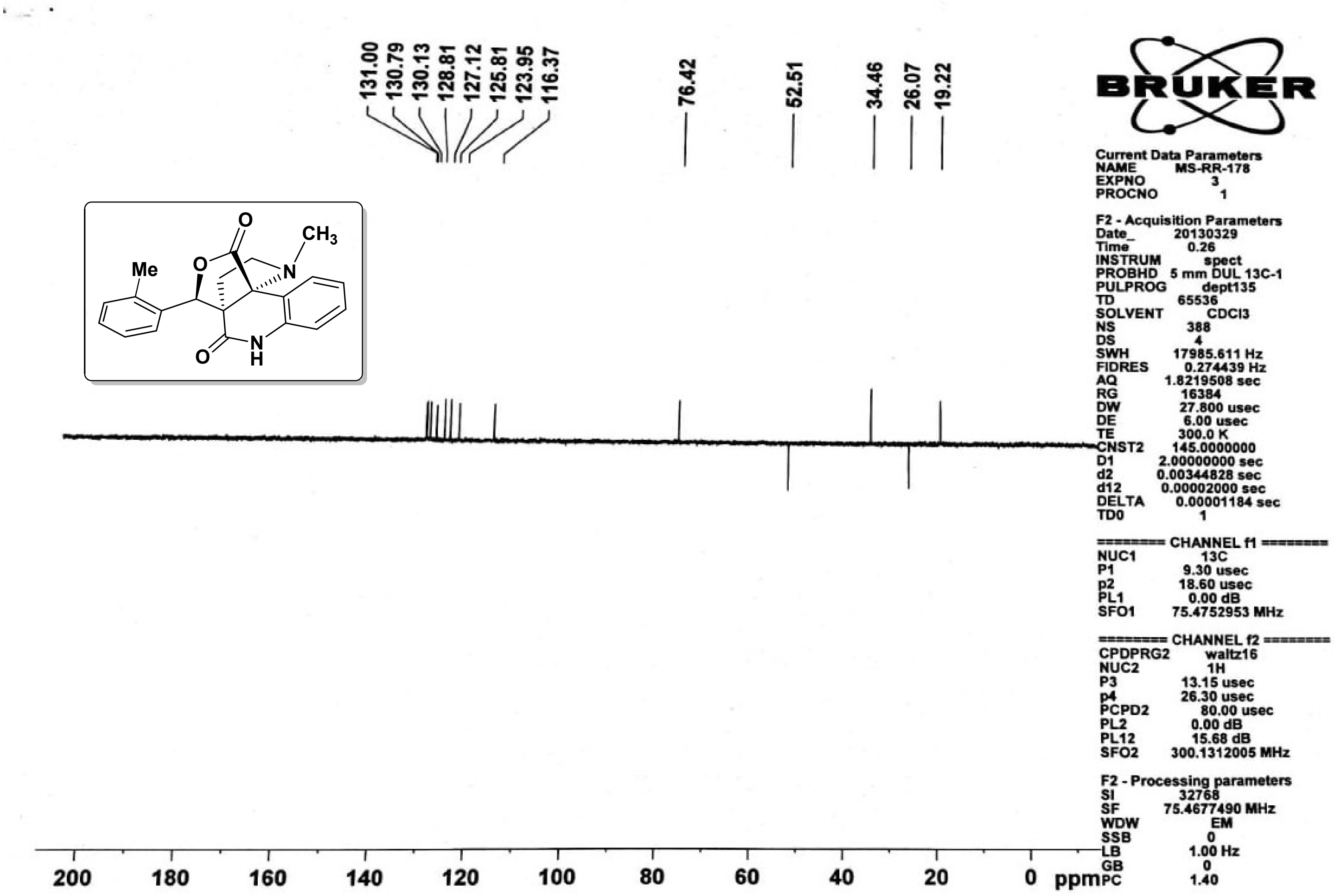

DEPT 135-NMR spectrum of compound **10b**

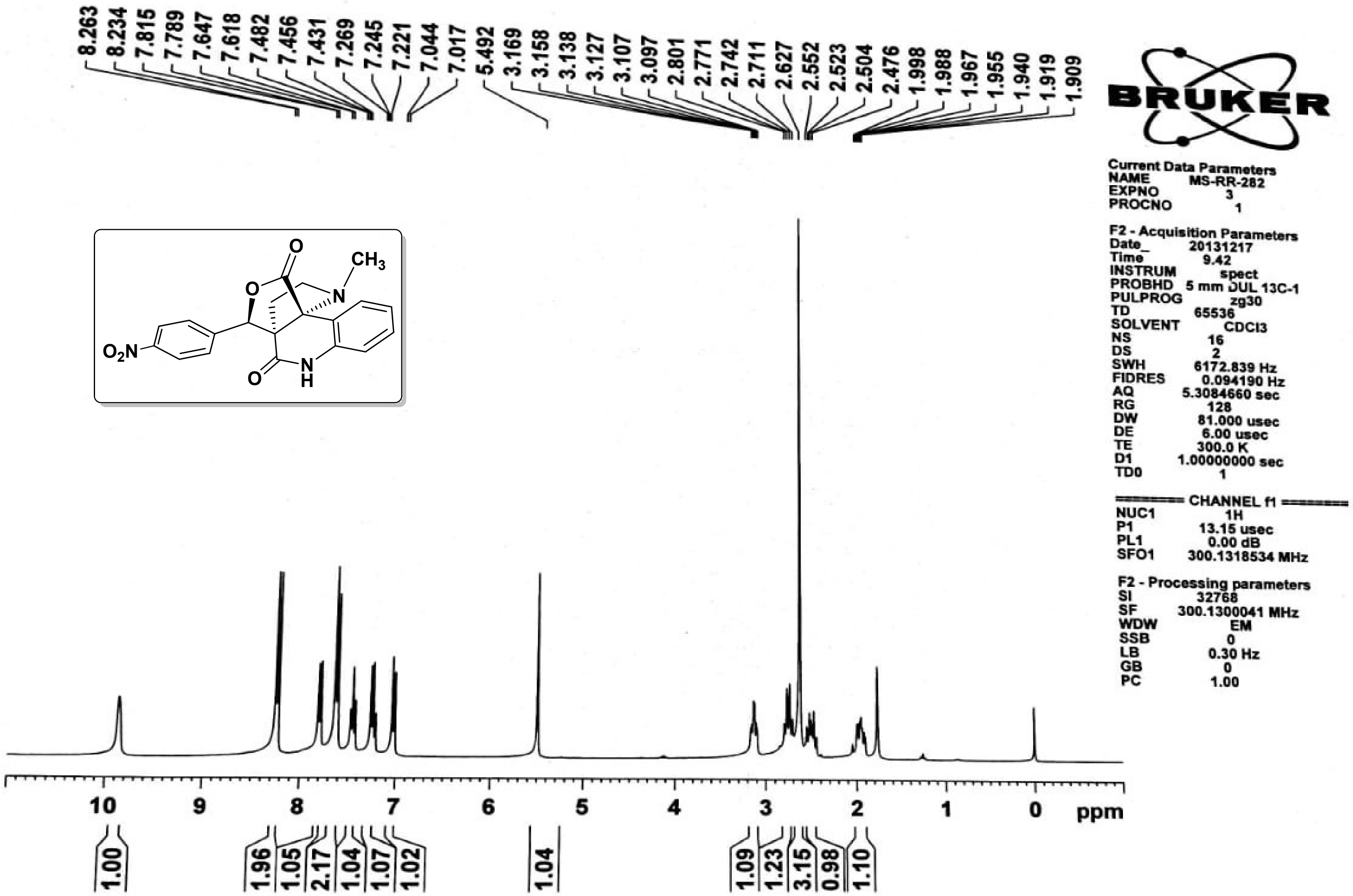

^{1}H NMR spectrum of compound **10c**

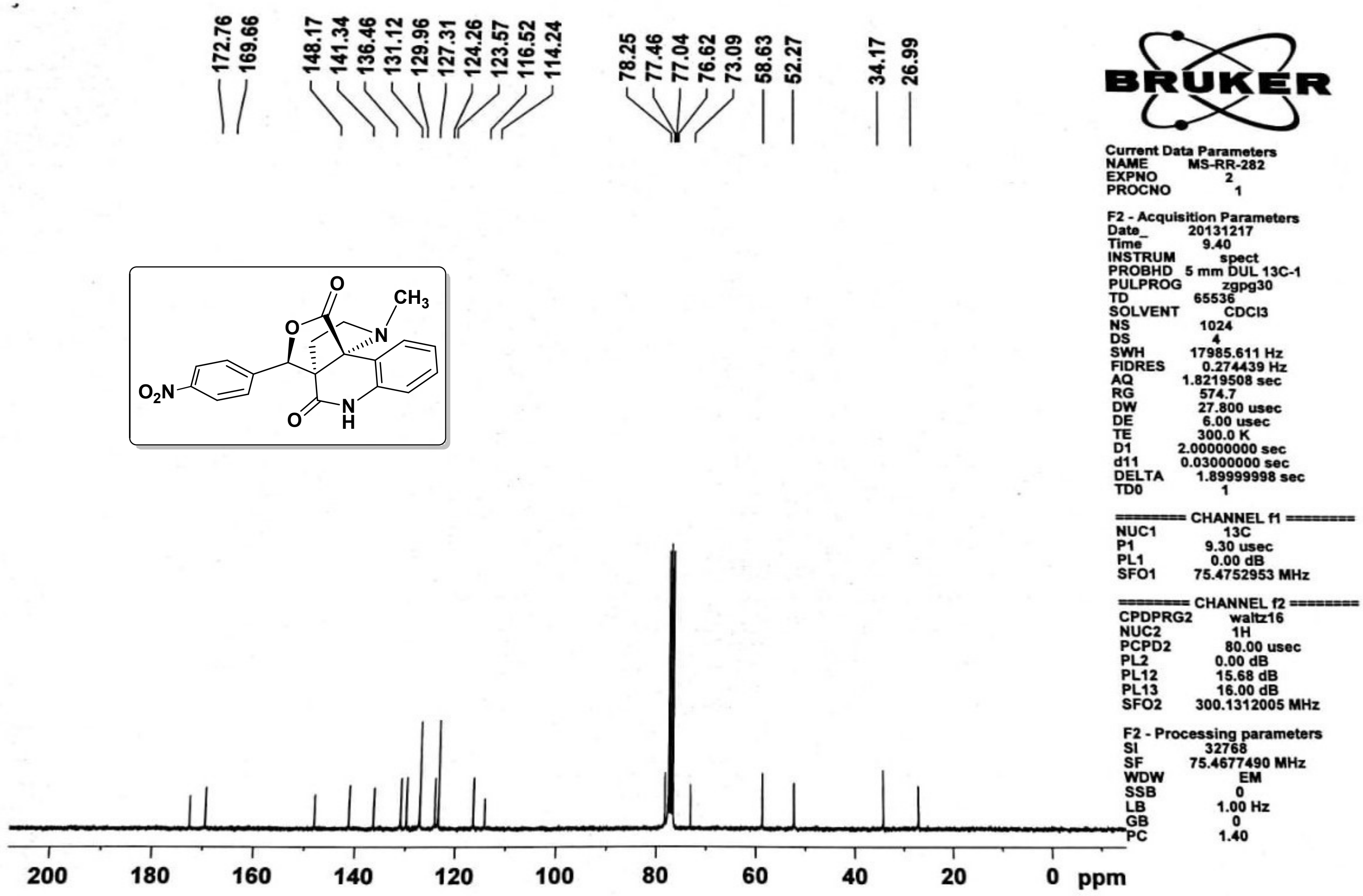

^{13}C-NMR spectrum of compound **10c**

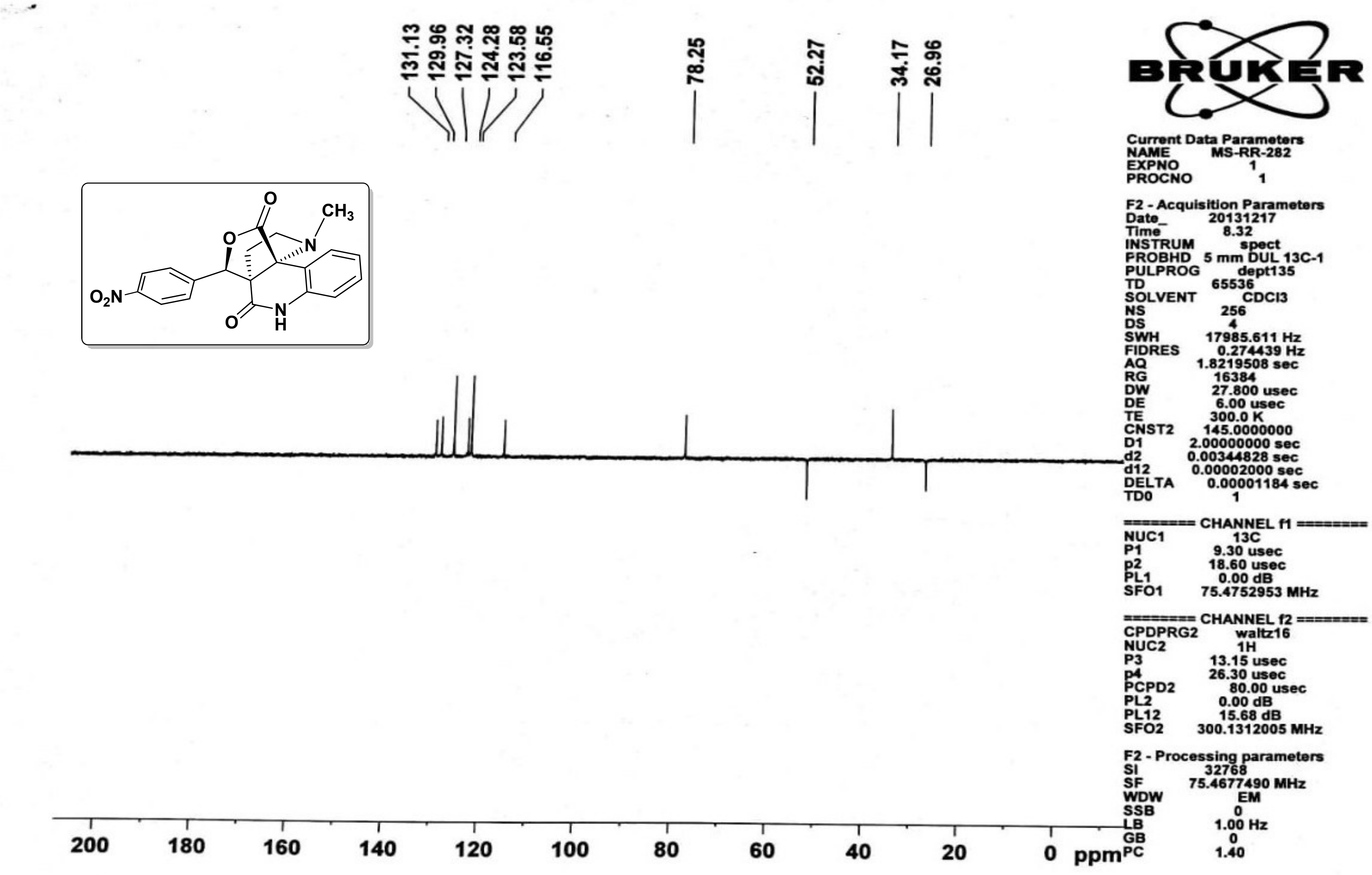

DEPT 135-NMR Spectrum of compound **10c**

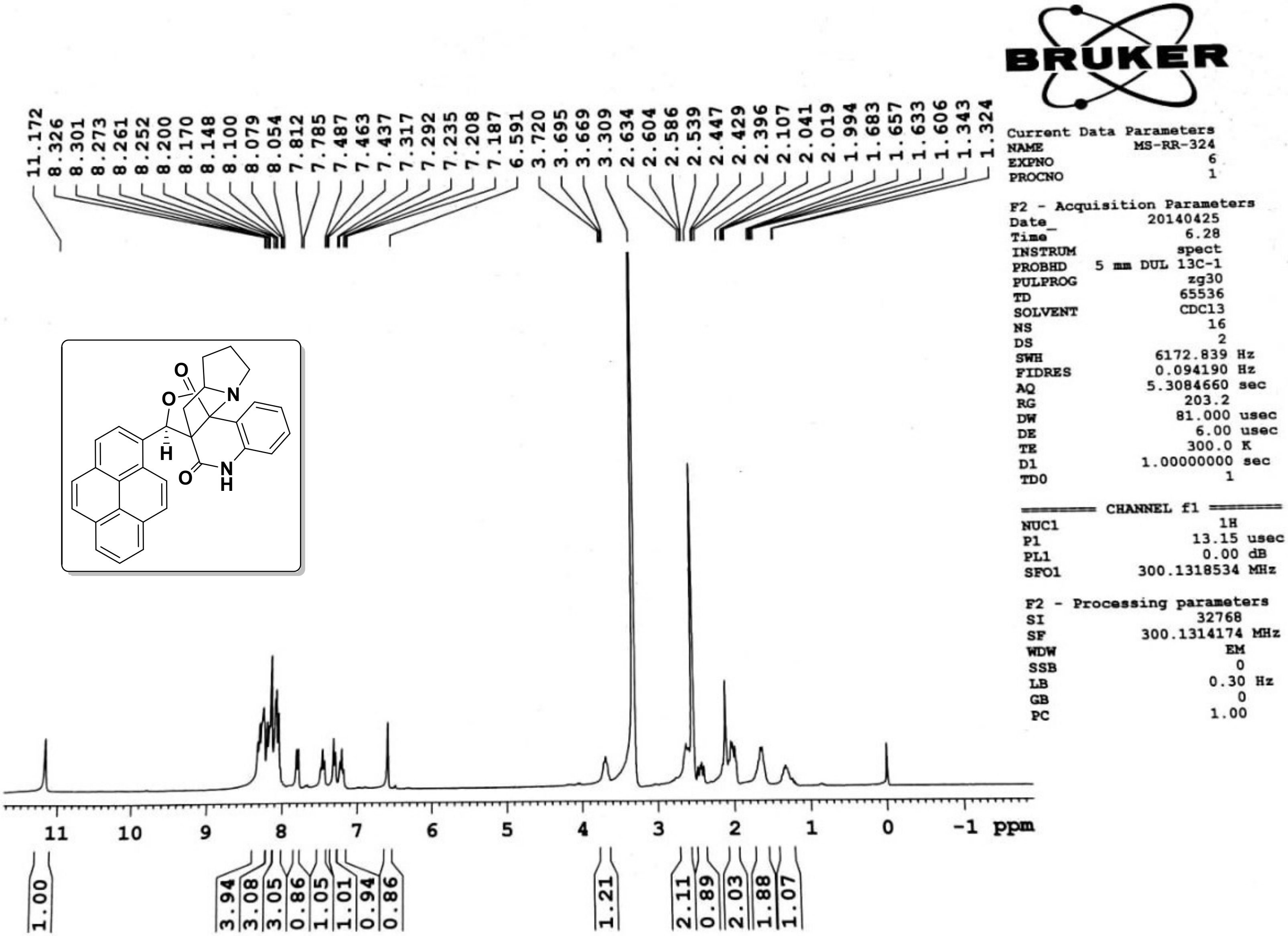

^{1}H NMR spectrum of compound **12a**

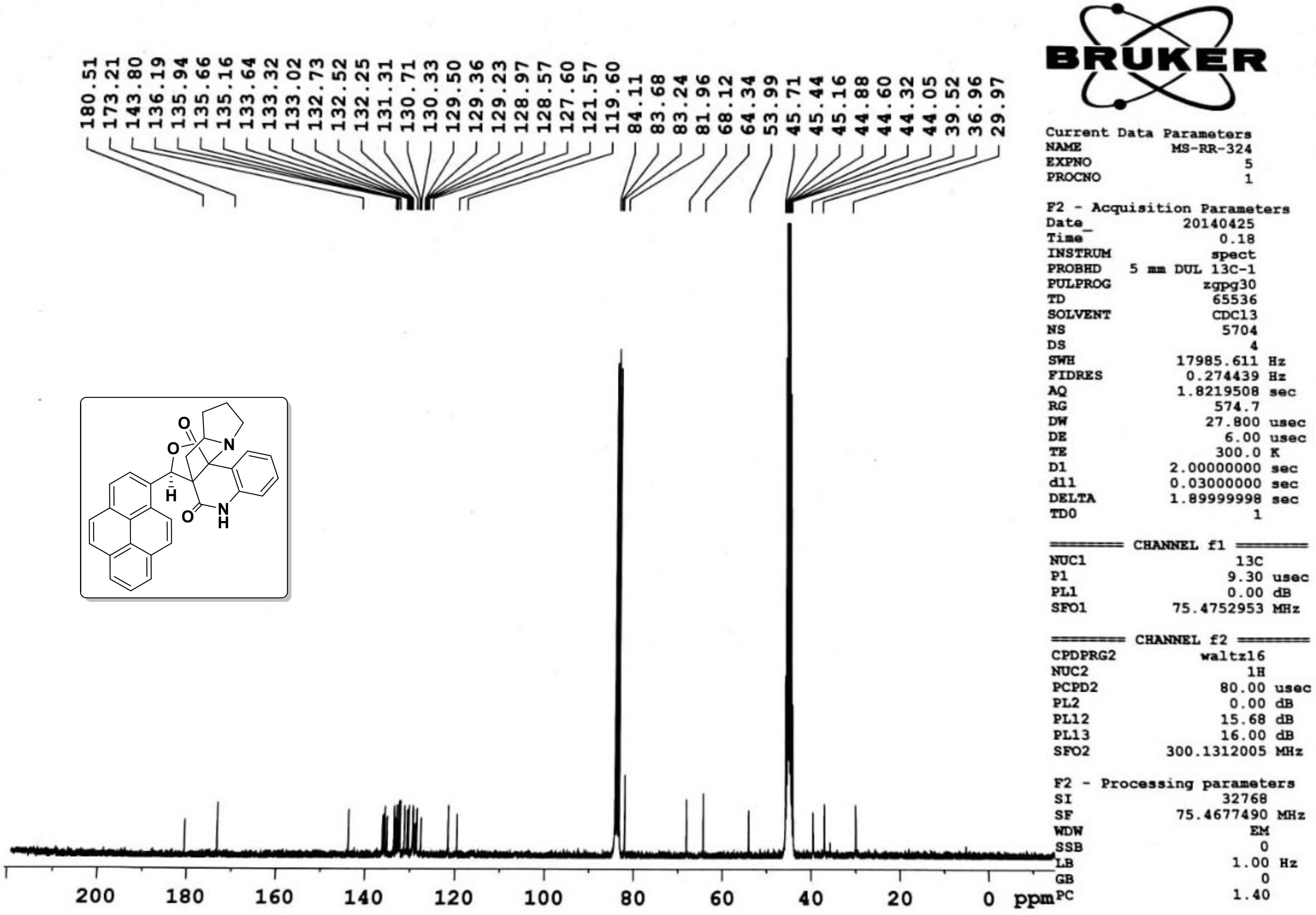

^{13}C NMR spectrum of compound **12a**

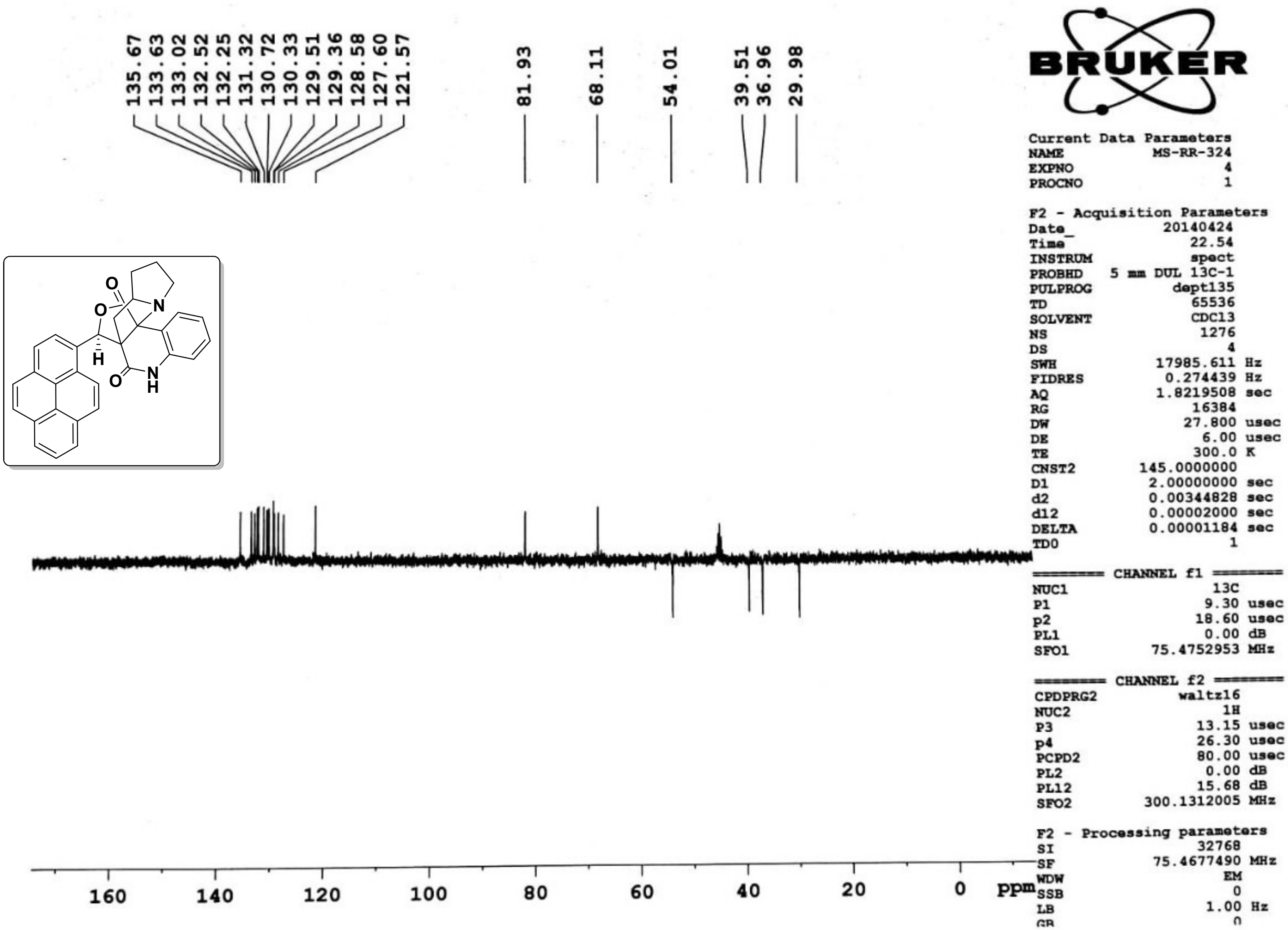

DEPT 135 NMR spectrum of compound **12a**

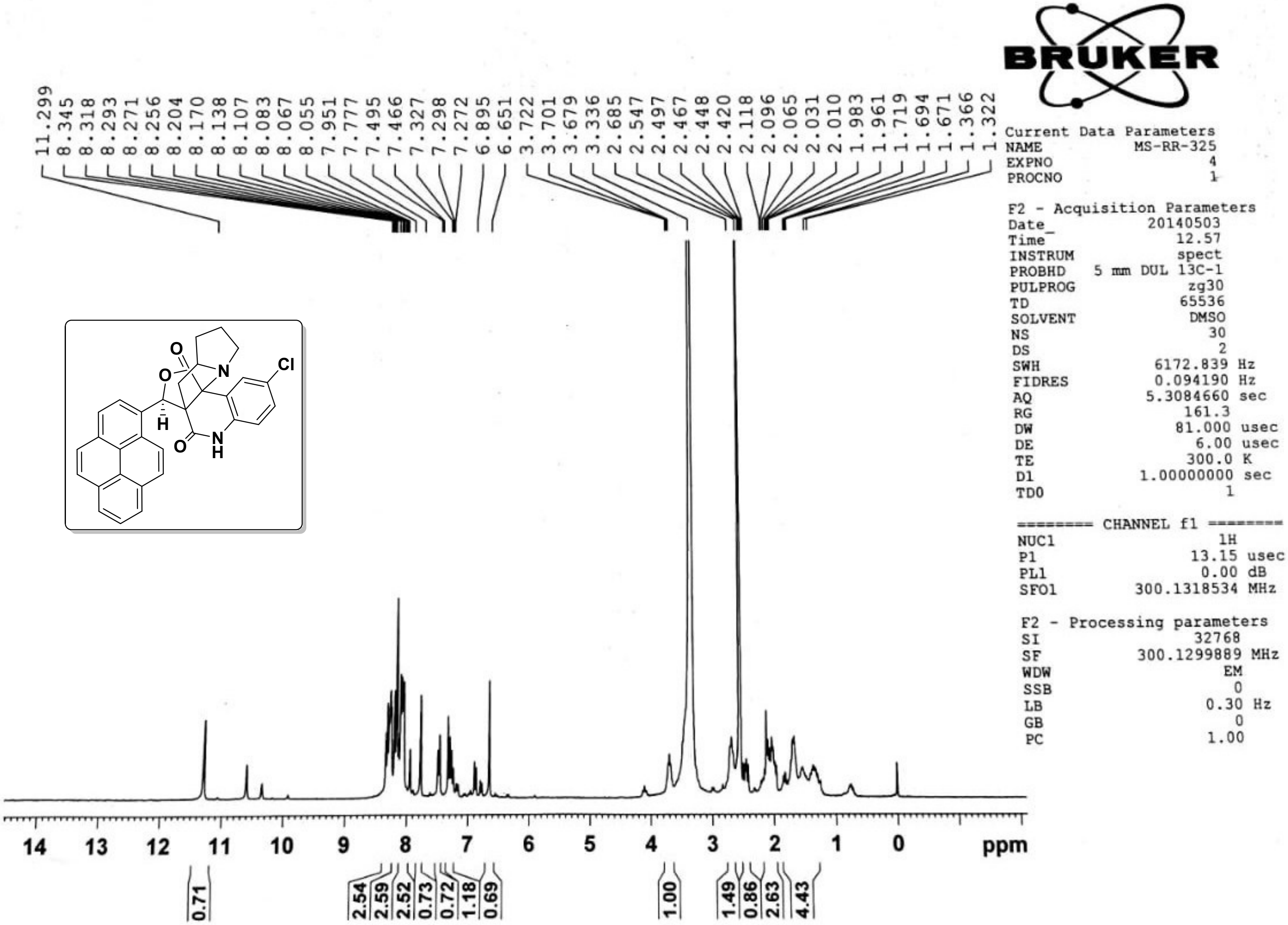

^{1}H NMR spectrum of compound **12b**

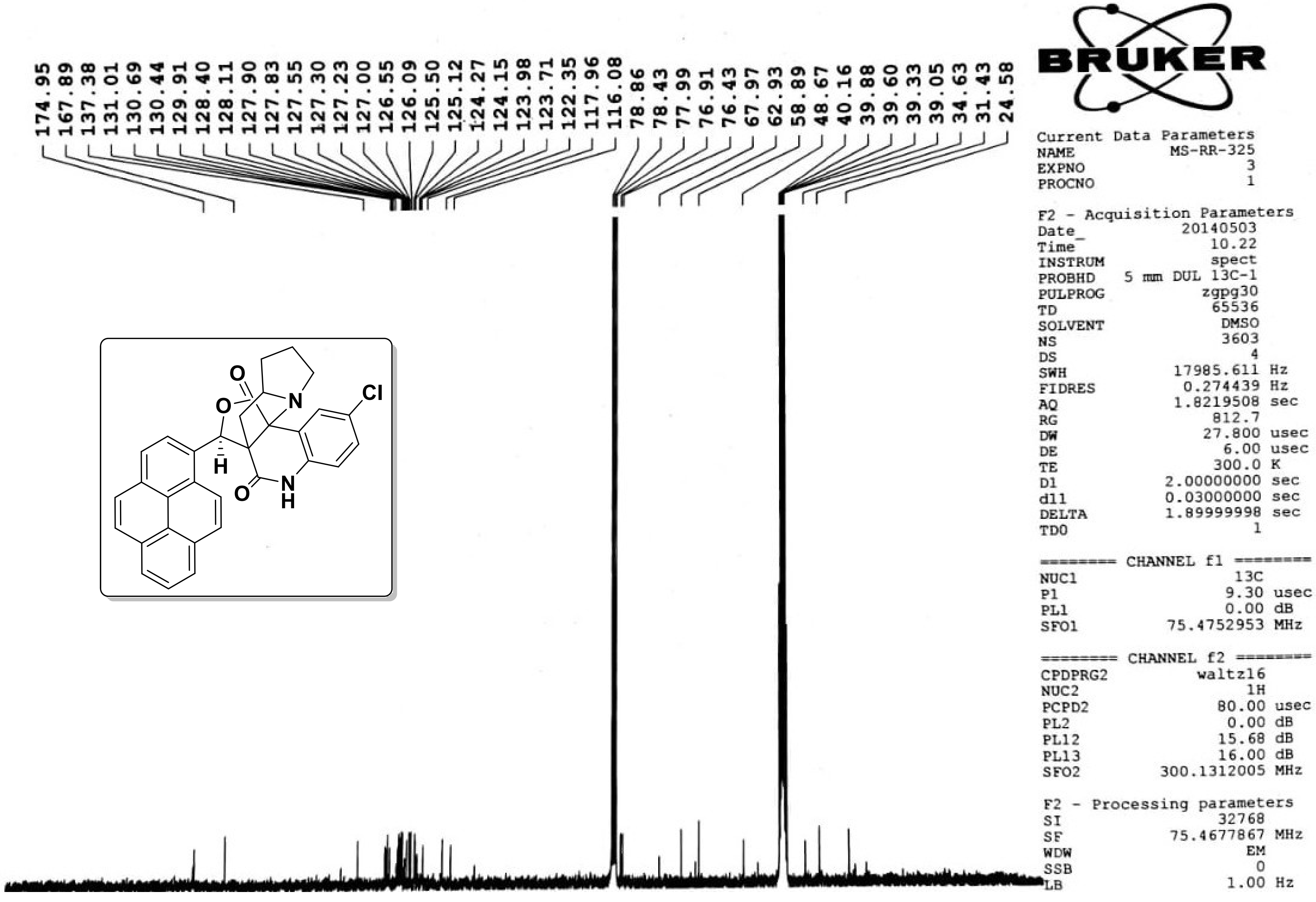

^{13}C NMR spectrum of compound **12b**

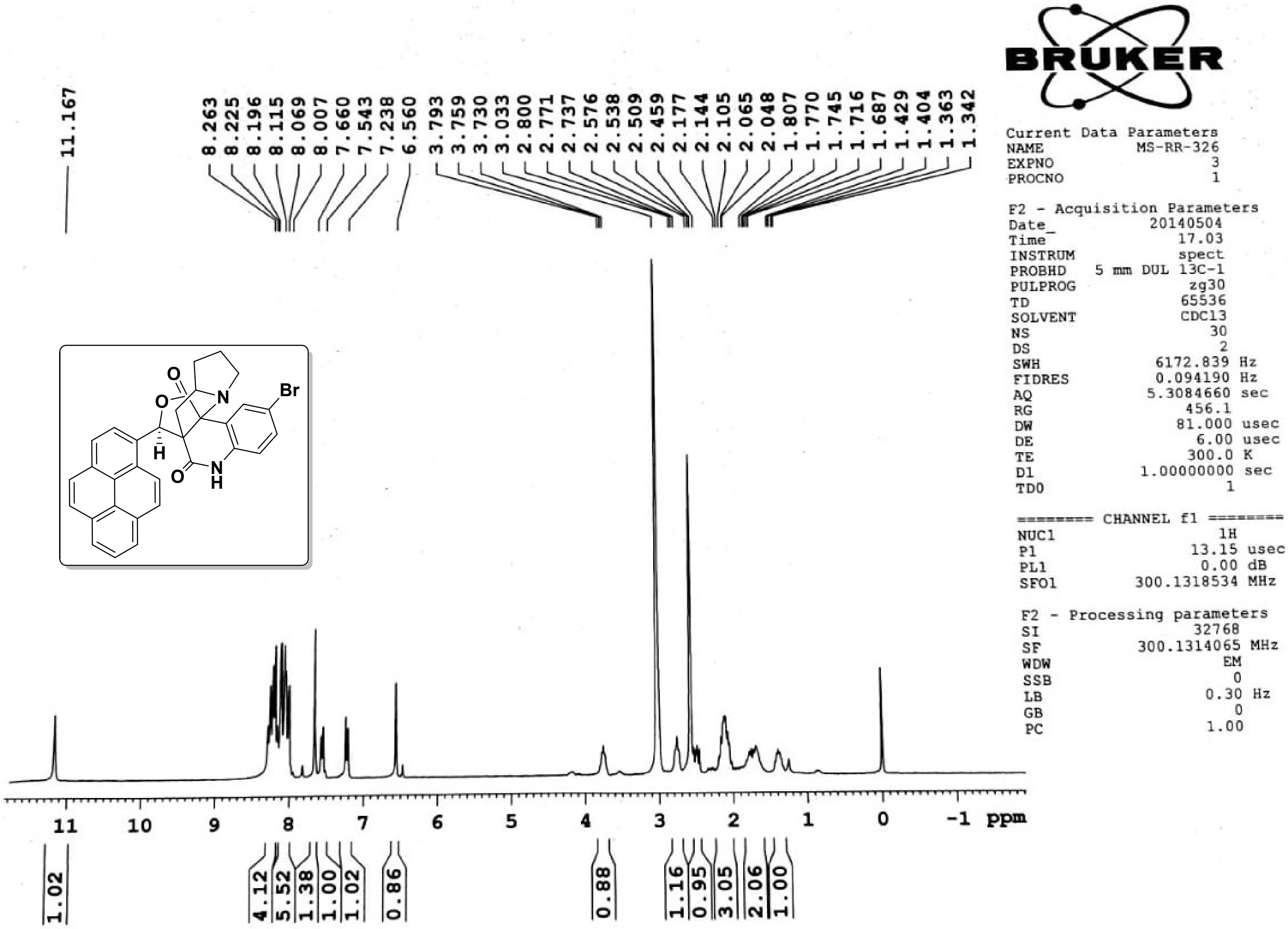

^{1}H NMR spectrum of compound **12c**

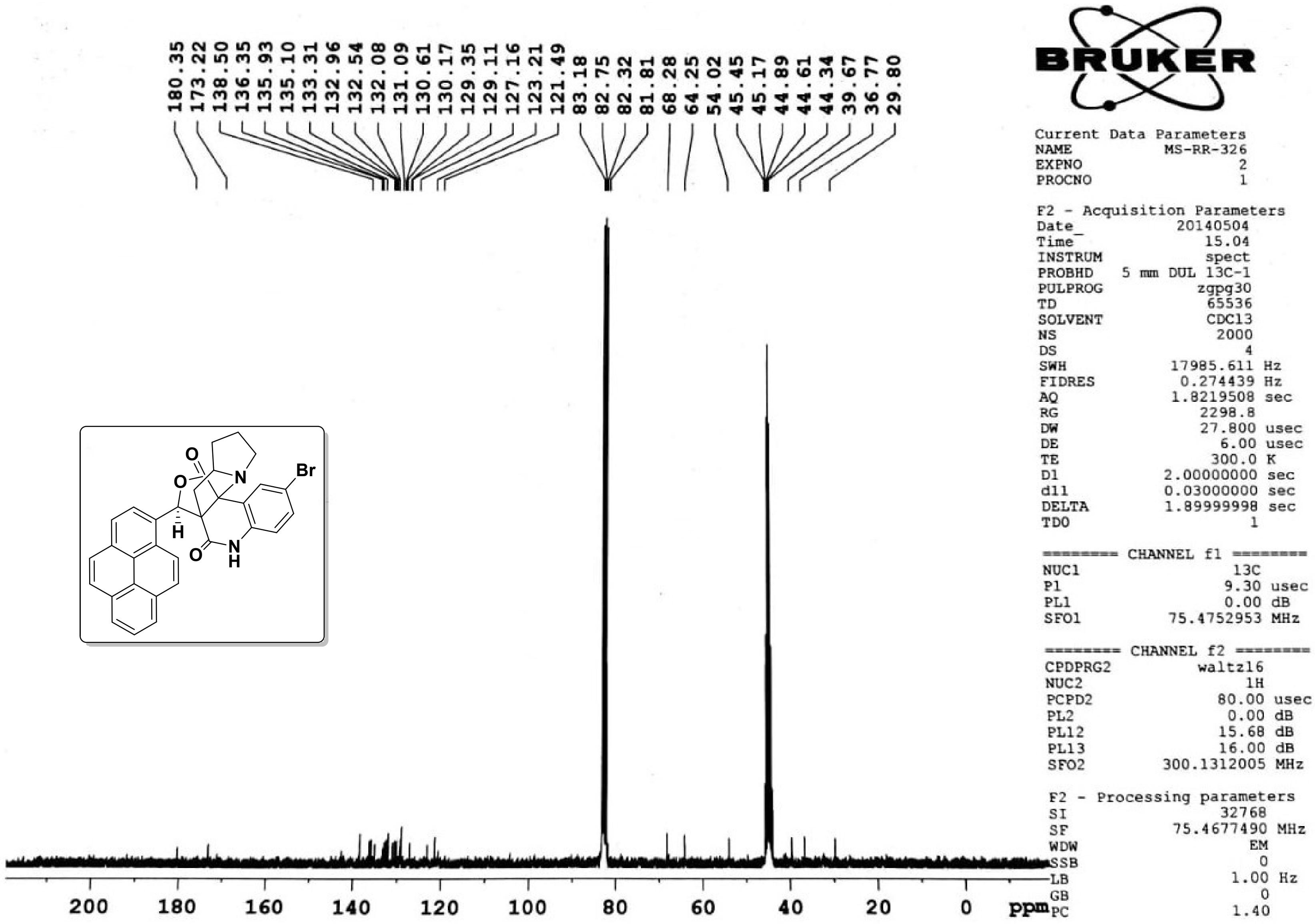

^{13}C NMR spectrum of compound **12c**

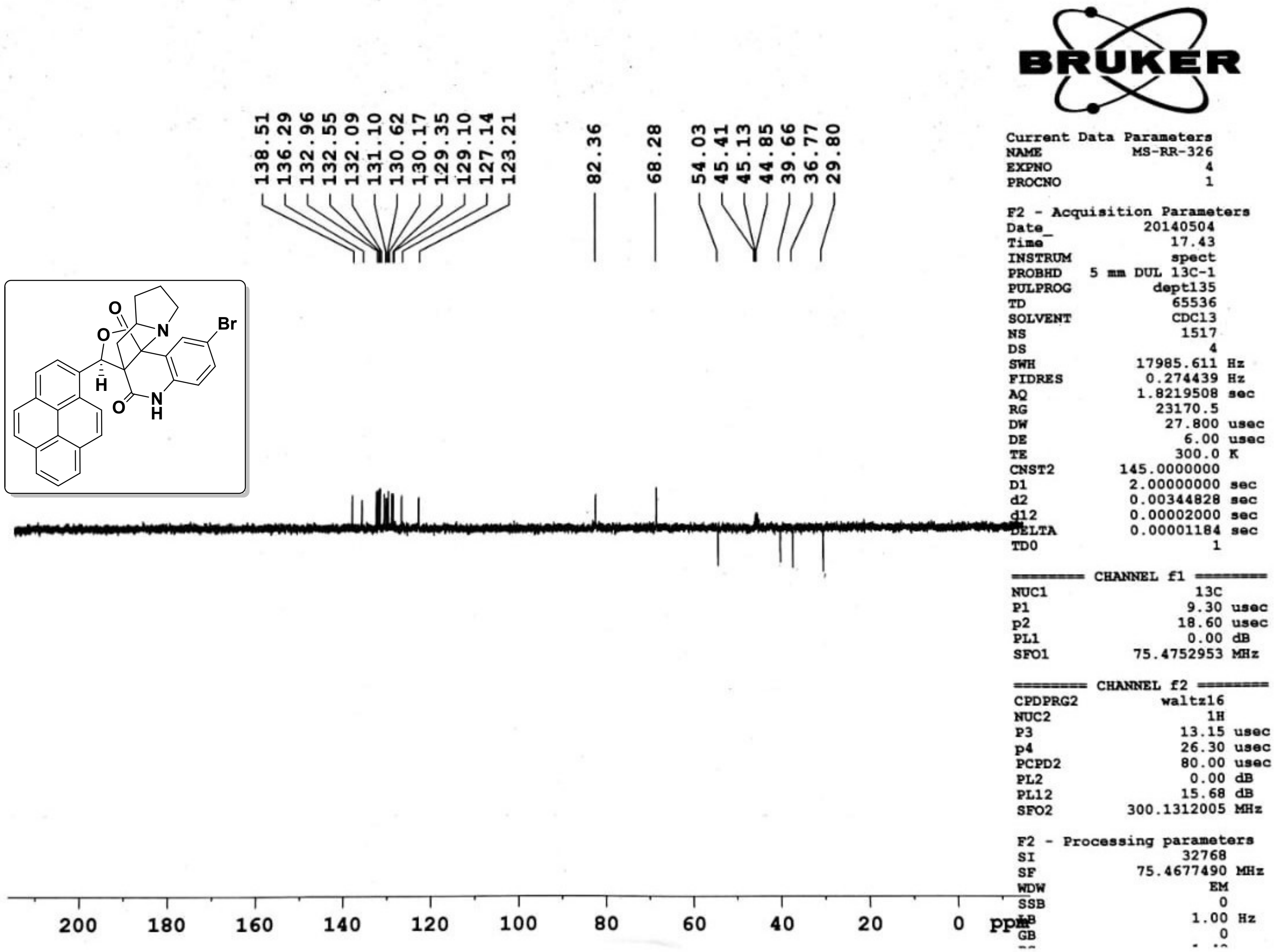

DEPT 135 NMR spectrum of compound **12c**
